# Examining the independent and moderating effects of arterial stiffness and cerebral blood flow on total hippocampal and hippocampal subfield volumes

**DOI:** 10.3389/fnagi.2025.1466294

**Published:** 2025-06-30

**Authors:** Michelle Horan, Daniel Carey, Silvin Knight, A. Fagan, James F. M. Meaney, Rose Anne Kenny, Céline De Looze

**Affiliations:** ^1^The Thomas Mitchell Centre for Advanced Medical Imaging (CAMI), St. James’ Hospital, Dublin, Ireland; ^2^School of Medicine, Trinity College Dublin, Dublin, Ireland; ^3^The Irish Longitudinal Study on Ageing (TILDA), Trinity College Dublin, Dublin, Ireland; ^4^Mercer’s Institute for Successful Ageing, St. James’ Hospital, Dublin, Ireland

**Keywords:** cognitive impairment, arterial stiffening, cerebral blood flow, hippocampus, hippocampal subfield atrophy

## Abstract

**Introduction:**

There is a critical link between vascular disease and the progression to dementia. The hippocampus has been implicated in memory and cognitive decline. In this study, we investigate the independent and moderating effects of increased arterial stiffness (AS) and reduced cerebral blood flow (CBF) on hippocampal volume (HV) in a large MRI sample of community-dwelling older adults from the Irish Longitudinal Study on Ageing (TILDA).

**Methods:**

Longitudinal data from study participants for Wave 1 (2009–2011) and Wave 3 (2014-2015) were included. This included health and social information as well as a nurse-administered health assessment. Patients who had complete AS, CBF and MR-hippocampal measurements were included. Pseudo-continuous arterial spine labelling was performed to quantify whole CBF. Volumetric analysis was performed using FreeSurfer 6.0 recon-all processing pipeline.

**Results:**

395 patients met inclusion criteria. This four-year follow up longitudinal study demonstrated that (i) prolonged elevated AS (at wave 1 and wave 3), (ii) the interaction between higher AS at wave 1 and lower CBF at wave 3 and (iii) the interaction between prolonged elevated AS (at wave 1 and wave 3) and reduced CBF at wave 3 were associated with smaller HV.

**Conclusion:**

Increased arterial stiffness and reduced CBF were not independently associated with smaller HV. However, in combination, persistently elevated AS and reduced CBF is associated with smaller HV. These effects were equally exerted across all hippocampal subfields tested. Our findings suggest a lag effect in the arterial stiffness and hippocampal volume relationship. We propose that the subsequent reduction in cerebral blood flow observed with elevated arterial stiffness may be the missing link in the pathway associating arterial stiffness with hippocampal atrophy.

## Introduction

Dementia is a major health issue with considerable health, social and economic costs for individuals, their families, and societies. It presents significant challenges to healthcare systems, affecting approximately 57 million people worldwide, a number which is predicted to rise to 153 million by 2050. As no cure exists, the identification of risk factors and the implementation of prevention strategies are a crucial step towards decreasing dementia incidence (Nichols et al., 2022). Cardiovascular health is believed to play an important role in this pathogenesis, and arterial stiffening has been identified as a key risk factor that increases dementia risk (Scuteri, 2007).

The deleterious effects of elevated arterial stiffness (AS) on cerebral health are well-established. Increased arterial stiffening has been associated with lower total brain volumes and a higher incidence of cerebral artefacts and white matter lesions in healthy older adults and in patient cohorts in both cross-sectional and longitudinal studies (Henskens et al., 2008; Matsumoto et al., 2007; Ohmine et al., 2008). The literature also supports a link between elevated arterial stiffness and cognitive impairment with progression to dementia (Henskens et al., 2008; Pase et al., 2016; Rouch et al., 2018; Benetos et al., 2012; Scuteri et al., 2007; Hanon et al., 2005). Negative associations between AS and total hippocampal volumes (a hallmark of AD) have been observed in many cross-sectional and longitudinal studies (Baradaran et al., 2021; Bown et al., 2021).

It has been proposed that increased arterial stiffness leads to cerebral hypoperfusion (Ajamu et al., 2021; Suri et al., 2020; Sadekova et al., 2018; Jefferson et al., 2018; Muhire et al., 2019; Tarumi et al., 2011). This may, in turn, lead to neuronal death with subsequent cerebral atrophy, as is seen selectively in the hippocampus in memory decline (Jack et al., 2000; Jack et al., 1998; Jobst et al., 1994). Several studies have reported an association between lower cerebral blood flow with smaller hippocampal volumes and progression to cognitive impairment (Knoops et al., 2009; Mak et al., 2014; Chao et al., 2010). The link between increasing arterial stiffness and reduced cerebral blood flow is also well established (Ajamu et al., 2021; Suri et al., 2020; Sadekova et al., 2018; Jefferson et al., 2018; Muhire et al., 2019; Tarumi et al., 2011). However, the causal relationships and interactions between these pathways, as well as associated compensatory mechanisms are less well understood. For example, to date, no studies have together described the independent and moderating effects of increased arterial stiffness and reduced CBF on the hippocampal volume in a healthy older population, but it could be assumed that raised arterial stiffness either alone or in combination with a reduction in cerebral blood flow may lead to selective atrophy of the hippocampus through this proposed mechanism of hypoperfusion and neuronal death. Additionally, no studies have addressed the issue of specific hippocampal subfields, and some subfields appear to be more vulnerable to hypoxia and ischemia than others, like the Cornu Ammonis (CA) 1, CA4, and subiculum subfields (Schmidt-Kastner and Freund, 1991; Bonnekoh et al., 1990; Kim et al., 2015). For example, a recent study by Pin et al. (2021), examined the rate of volume loss in the hippocampal subfields CA1-CA3, CA4 and the subiculum over four-years in 249 non-demented older adults and examined their interaction with white matter hyperintensities, believed to be a surrogate marker for vascular damage. They identified the subiculum to be particularly vulnerable to ischemia with a significant association between WMH’s and subiculum volume loss, independent of vascular risk factors (*p* = 0.03) (Pin et al., 2021).

In this study, we investigate the independent and moderating effects of AS and CBF on total hippocampal volume in a large MRI sample of community-dwelling older adults from a nationally representative population-based study, the Irish Longitudinal Study on Ageing (TILDA). In supplementary analyses, we also examine if these effects are specific to certain hippocampal subfields which are known to be vulnerable to AS and CBF alterations: the CA1, CA3, CA4, subiculum, and dentate gyrus (DG). Specifically, we test the hypotheses that (i) higher AS is associated with lower total hippocampal volumes and that (ii) reduced CBF may moderate this relationship. We also test the hypotheses that (iii) persistently higher AS over a four-year follow-up period and (iv) the co-occurrence of higher AS and low CBF may have more significant effects on hippocampal volumes. Finally we hypothesize that these effects may be exerted maximally in the CA-1, CA-4 and subiculum subfields.

## Methods

### Study design and participants

We used data from a nationally representative population-based study of community-dwelling older adults aged 50 years and older resident in the Republic of Ireland, The Irish Longitudinal Study on Ageing (TILDA). TILDA’s random sampling procedure and study design have been previously described (Whelan and Savva, 2013). Participants have been followed biennially from Wave 1 (2009–2011), and this study includes data from Wave 1 (2009–2011) and Wave 3 (2014–2015). At each wave, a computer-assisted personal interview (CAPI) collected information on the participants’ social and health information. At Wave 1 and Wave 3, participants were also invited to take part in an extensive nurse-administered health assessment in a dedicated health centre or a modified version in the participant’s home. Among the 6,687 who completed a CAPI at Wave 3, 4,309 attended the health centre, of whom 560 underwent multimodal Magnetic Resonance Imaging (MRI). The study sample consists of 395 participants who had arterial stiffness measurements at Wave 1 and Wave 3, and cerebral blood flow and MR-hippocampal volume measurements at Wave 3 (Supplementary Figure 1).

### MRI protocol

MRI scanning was completed at Wave 3, at the National Centre for Advanced Medical Imaging (CAMI) at St. James’s Hospital, Dublin, using a 3 Tesla Philip’s Achieva system and a 32 channel head coil. The protocol included a T1-weighted 3D Magnetisation Prepared Rapid Gradient Echo (MP-RAGE) sequence, with the following parameters: FOV (mm): 240 × 218 × 162; 0.9 mm isotropic resolution; SENSE factor: 2; TR: 6.7 ms; TE: 3.1 ms; flip angle: 9; and pseudo-continuous arterial spin labelling (pCASL) sequences. Upstreaming arterial blood flow in the neck was labelled using a series of tightly packed inversion pulses. Following this, a short interval was allowed to facilitate this labelled blood to perfuse the brain parenchyma. The perfused brain was then imaged using a 2D multislice single echo-planar imaging (EPI) with suppression of the background tissue performed on two occasions during the pCASL sequence. The pCASL sequences parameters were: field of view 240 × 240 mm^2^, the matrix was 80 × 80, the repetition time was 4,000 ms, the echo time was 9 ms, flip angle of 90^o^, SENSE of 3.5 and the scan duration was 4 min and 16 s. A total of 13 slices were obtained yielding 30 dynamic scans and a total of 780 images. An 1800 millisecond labelling duration in addition to a post-label delay (PLD) of 1800 ms were applied, as advised in the ASL consensus paper (Alsop et al., 2015).

### Outcome

#### Hippocampal volume measurement

All T1w images were reviewed by a trained operator blind to participant identity for image artifact and for the presence of lesions. Volumetric analysis was performed using FreeSurfer version 6.0 recon-all processing pipeline, an automated segmentation tool for volume measurement (Dale et al., 1999; Fischl et al., 1999). The details of the segmentation methods have been previously described (Iglesias et al., 2015). The hippocampal subfield option, described previously (Iglesias et al., 2015), was used to segment the hippocampus and its subfields and we extracted the CA-1, CA-3, CA-4, subiculum and dentate gyrus (DG). All hippocampal segmentations were reviewed by a trained operator who was blinded to participant identity and no datasets had significant error reported by this trained operator in both total and subfield volume measurements.

### Exposures

#### Arterial stiffness (AS)

Carotid-femoral pulse wave velocity (cf-PWV) was measured at Wave 1 and Wave 3 (at four-year follow up) by tonometry (Vicorder®). This non-invasive measurement is the gold standard for quantifying arterial stiffness and is performed by measuring the pulse wave velocity at the carotid artery and the femoral artery. In TILDA, mean cf-PWV was calculated at each wave as the average of two cf-PWV measurements. A cf-PWV value greater than 12 m/s is indicative of arterial stiffness, as outlined by the European Society of Hypertension and European Society of Cardiology (2013).

#### Total cerebral blood flow (CBF)

Mean whole brain grey matter cerebral blood flow (CBF) at Wave 3 was quantified as the rate of delivery of arterial blood to brain tissue measured using pCASL-MRI. MRI analysis was performed using Oxford ASL (v.4.0) (*FMRIB Software Library, FSL*; The University of Oxford, UK) in the FMRIB Software Library (Smith et al., 2004). Analyses steps have been described elsewhere (Knight et al., 2021).

### Covariates

Covariates included age, sex, education (none/primary, secondary, or tertiary/higher), pre-existing self-reported physician-diagnosed cardiovascular diseases and events (CVDEs), systolic and diastolic blood pressure, antihypertensive medications, lifestyle factors and estimated intracranial volume (eTIV). These were identified as potential confounders of the relationships between AS, CBF and hippocampal volumes. CVDEs included history of angina, heart attack, congestive heart failure, stroke, transient ischemic attack, and atrial fibrillation. Data were pooled to create a dichotomous CVDEs variable, for absence (disease-free) or presence (≥1) of CVDEs. Average seated blood pressure was measured using an Omron blood pressure monitor (two readings taken 1 min apart). Antihypertensive medications corresponded to the anatomical therapeutic chemical codes C02, C03, C07, C08, or C09. Smoker status was defined as never, past, or current. The CAGE questionnaire (Mayfield et al., 1974) was used as the measure of problematic alcohol consumption and was represented as a binary variable. Physical activity was recorded through the International Physical Activity Questionnaire (IPAQ) (Hagströmer et al., 2006); short-form and classified as low, moderate or high. BMI: weight and height were measured using a SECA electronic floor scales and a SECA 240 wall mounted measuring rod, respectively.

### Statistical analysis

#### Data descriptors

All statistical analyses were performed using R software version 4.2.2 (R Core Team, 2020). Descriptive statistics are given for the study sample and per AS group (cf-PWV ≤ 12 m/s vs. cf-PWV > 12 m/s) and CBF tertile. Continuous variables are described as unadjusted means with standard deviations (SD); categorical variables are given as percentages (%). Independent *t*-tests and chi-squared tests were used to assess differences between AS groups; ordered logistic regressions were used to assess differences between tertiles of CBF. To estimate selection bias, the observed sample was also compared to the excluded cohort using independent *t*-tests and chi-squared tests.

#### Examining the relationship between AS, CBF, and total hippocampal volume

Ordinary Least Squares (OLS) regressions were used to assess the independent and moderating effects of AS and CBF on total hippocampal volume. Cross-sectional analyses included *Cf*-PWV at Wave 3 and CBF at Wave 3 (continuous variables) as predictors in separate models; they were then adjusted for each other in subsequent models; final models included an interaction term between the two. Further analyses included *Cf*-PWV at Wave 1 as predictor. Therefore, final models also included an interaction term between *Cf*-PWV at Wave 1 and *Cf*-PWV at Wave 3 and between *Cf*-PWV at Wave 1, *Cf*-PWV at Wave 3 and CBF at Wave 3.

#### Supplementary analyses

Linear mixed effect models were used to examine the independent and moderating effects of AS and CBF on hippocampal subfield volumes. Subfield volumes were z-score transformed to allow direct comparison of the effects of AS and CBF by subfield, as the volume distribution of each subfield varies. In cross-sectional analyses, fixed effects included, in separate models first, *Cf*-PWV and CBF at Wave 3 and hippocampal subfields (the CA1 subfield set as the reference level) with an interaction term. *Cf*-PWV x subfields and CBF x subfields were then adjusted for each other in subsequent models. Finally, three way interactions between *Cf*-PWV, CBF and subfield volumes were tested. In follow-up analyses, fixed effects were *Cf*-PWV at Wave 1 and hippocampal subfields with an interaction term. In subsequent models, *Cf*-PWV at Wave 1 x subfield volumes was adjusted for *Cf*-PWV at Wave 3 x subfield volumes and for CBF at Wave 3 x subfield volumes. Final models included three way interactions between *Cf*-PWV at Wave 1, *Cf*-PWV at Wave 3 and subfield volumes, and between *Cf*-PWV at Wave 1, CBF at Wave 3 and subfield volumes. We also tested a four way interaction between *Cf*-PWV at Wave 1, *Cf*-PWV at Wave 3, CBF and subfield volumes. The significance of interaction terms was assessed through likelihood ratio tests comparing additive models with models with an interaction term. In all models, participants constituted the random intercept. In all analyses, baseline models included age, sex and eTIV as covariates. Fully adjusted models further controlled for education, CVDEs, blood pressure, antihypertensive medications, and lifestyle factors.

## Results

### Sample characteristics

A total of 395 participants were included in our analysis (Supplemental Figure 1). Mean age was 68.3 years (SD 7.2), 54.2% were women (Table 1). Compared to the excluded cohort (*n* = 6,292), the included participants were older (*p* < 0.01), more likely to have a higher level of education (*p* < 0.01), to be non-smoker (*p* < 0.01), have a lower BMI (*p* = 0.04) and more likely to exercise (*p* = 0.02).

**Table 1:** Characteristics of the study sample (*N* = 395).

Characteristics	Study sample *N* = 395 (SD)
Age (years), mean (SD)	68.3 (7.2)
Sex (Female, %)	54.2
Education (Low, %)	20.2
CVD conditions (>1, %)	61.5
Systolic/ Diastolic BP (mean, sd) mmHg	133.9 (18.9) / 79.8 (10.2)
Antihypertensives (on meds, %)	14.6
BMI (mean, sd) kg/m^2^	27.9 (4.3)
Smoking (Present, %)	5.3
Physical activity (low, %)	33.6
Problematic alcohol (%)	8.8
*Cf*-PWV at W1 m/s	10.7 (1.9)
*Cf*-PWV at W3 m/s	10.8 (2.1)
CBF ml/100 g/min	36.5 (7.9)

At Wave 1, n = 50 (13%) of the cohort had a cf-PWV value of greater than 12 m/s. Similar prevalence was observed at Wave 3 with 58 participants (15%) having raised arterial stiffness. Mean CBF was 36.5 (SD = 7.9) and no difference in mean CBF was found between cf-PWV groups (cf-PWV ≤ 12 m/s vs. cf-PWV > 12 m/s) at Wave 1 (*p* = 0.80) or Wave 3 (*p* = 0.34). Descriptive characteristics per AS group at Wave 1 and Wave 3 and CBF tertile at Wave 3 are given in Supplementary Tables 1–3.

### The independent and moderating effects of AS and CBF on total hippocampal volume

#### Cross-sectional analyses

In baseline models, no statistically significant association was seen between *Cf*-PWV (*B* = 0.04; 95% CI = −0.01, 0.07; *p* = 0.06) or CBF (*B* = 0.01; 95% CI = −2.45 × 10^−3^, 0.02; *p* = 0.19) at Wave 3 and total hippocampal volume when examined independently or when mutually adjusted including an interaction term between *Cf*-PWV and CBF which also did not reach statistical significance (*B* = 3.70 × 10^−3^; 95% CI = −1.07 × 10^−3^, 8.47 × 10^−3^; *p* = 0.09). Findings were the same in fully adjusted models (Supplementary Table 4).

#### Follow-up analyses

**Figure 1:**
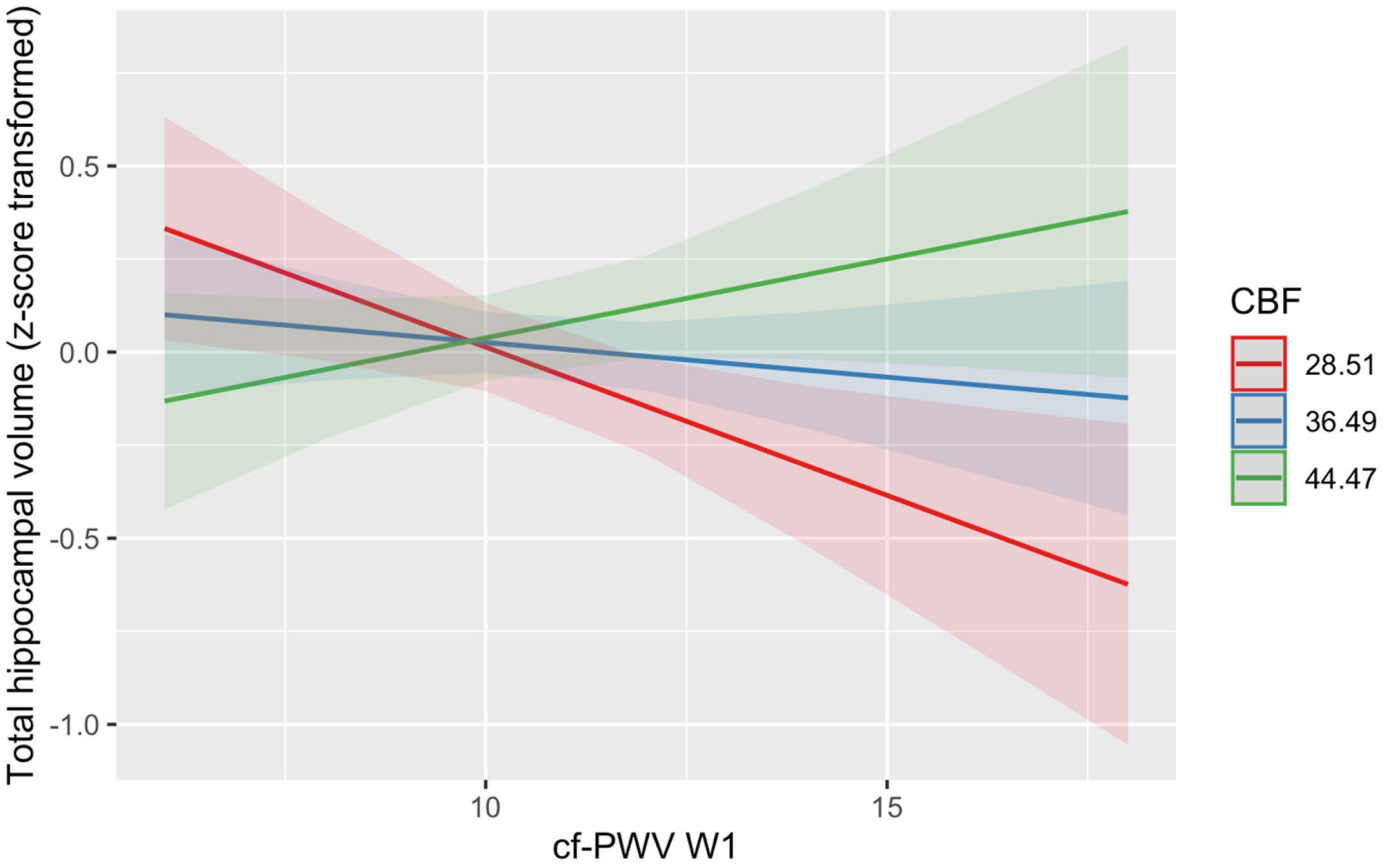
The combined effects of *Cf*-PWV at Wave 1 and CBF at Wave 3 on total hippocampal volume. *Cf*-PWV = Carotid-femoral pulse wave velocity (m/s); W1/3 = Wave 1/3; CBF_GM =_ Mean whole brain grey matter cerebral blood flow (ml/100 g/min). The interaction between *Cf*-PWV at Wave 1 and CBF at Wave 3 was also significant (*p* = 0.003); the combination of high *Cf*-PWV at Wave 1 and low CBF at Wave 3 was associated with lower total hippocampal volume, suggesting that high CBF may serve as buffer against volume atrophy in the presence of high cf-PWV (i.e raised arterial stiffness).

In baseline models, no significant association between *Cf*-PWV at Wave 1 and total hippocampal volume (B = -0.02; 95% CI = −0.06, 0.02; *p* = 0.37) was seen when examined alone or when adjusted for CBF in an additive model (B = -0.02; 95% CI = −0.06, 0.02; *p* = 0.38). However, a model including an interaction between *Cf*-PWV at Wave 1 and CBF shows a statistically significant interaction (B = 0.01; 95% CI = 2.50 × 10^−3^, 0.01; *p* < 0.01; Table 2, Panel B, Figure 1); the interaction between higher *Cf*-PWV at Wave 1 and lower CBF at Wave 3 was associated with lower hippocampal volume, suggesting that high CBF may serve as buffer against volume atrophy in the presence of high AS.

**Table 2:** Estimates and 95% confidence intervals of the combined effects of Carotid-femoral pulse wave velocity (*Cf*-PWV) i.e. arterial stiffness at Wave 1 and cf-PWV at Wave 3 (Panel A), the combined effects of *Cf*-PWV at Wave 1 and cerebral blood flow (CBF) at Wave 3 (Panel B) on total hippocampal volume and the three-way interaction model of cf-PWV at wave 1, cf-PWV at wave 3 and CBF at wave 3 on total hippocampal volumes (Panel C).

Variables	Panel A: combined effects of cf-PWV Wave 1 and cf-PWV-Wave 3 on total hippo volume		Panel B: combined effects of cf-PWV Wave 1 and CBF-Wave 3 on total hippo volume		Panel C: three-way interactions of cf-PWV Wave 1, cf-PWV Wave 3 and CBF Wave 3 on total hippocampal volumes.	
	Estimates (95% CI)	*p*-value	Estimates (95% CI)	*p*-value	Estimates (95% CI)	*p*-value
PWV-W1	0.14 (−0.04, 0.32)	0.12	−0.30 (−0.49, −0.11)	0.002**	1.92 (0.92, 2.92)	<0.001***
PWV-W3	0.25 (0.07, 0.43)	0.007**	-	-	2.15 (1.21, 3.09)	<0.001***
CBF_W3	-	-	−0.07 (−0.13, −0.02)	0.007**	0.51 (0.24, 0.78)	<0.001***
PWV-W1 xPWV-W3	−0.02 (−0.02, −0.002)	0.03*	-	-	−0.19 (−0.28, −0.11)	<0.001***
PWV-W1 x CBF-W3	-	-	0.01 (0.003, 0.01)	0.003**	−0.05 (−0.07, −0.02)	<0.001***
PWV-W3 x CBF-W3	-	-	-	-	−0.05 (−0.08, −0.03)	<0.001***
PWV-W1 x PWV-W3 x CBF-W3	-	-	-	-	0.005 (0.003, 0.007)	<0.001***
Covariates						
Age	−0.07 (−0.08, −0.06)	<0.001***	−0.06 (−0.08, −0.05)	<0.001***	−0.07 (−0.08, −0.06)	<0.001***
Sex	−0.33 (−0.53, −0.14)	<0.001***	−0.39 (−0.59, −0.20)	<0.001***	−0.33 (−0.52, −0.14)	<0.001***
eTIV	0.37 (0.27, 0.47)	<0.001***	0.37 (0.28, 0.47)	<0.001***	0.38 (0.29, 0.48)	<0.001***

***p < 0.001, **p < 0.01 and *p < 0.05 indicate significant differences.

**Figure 2:**
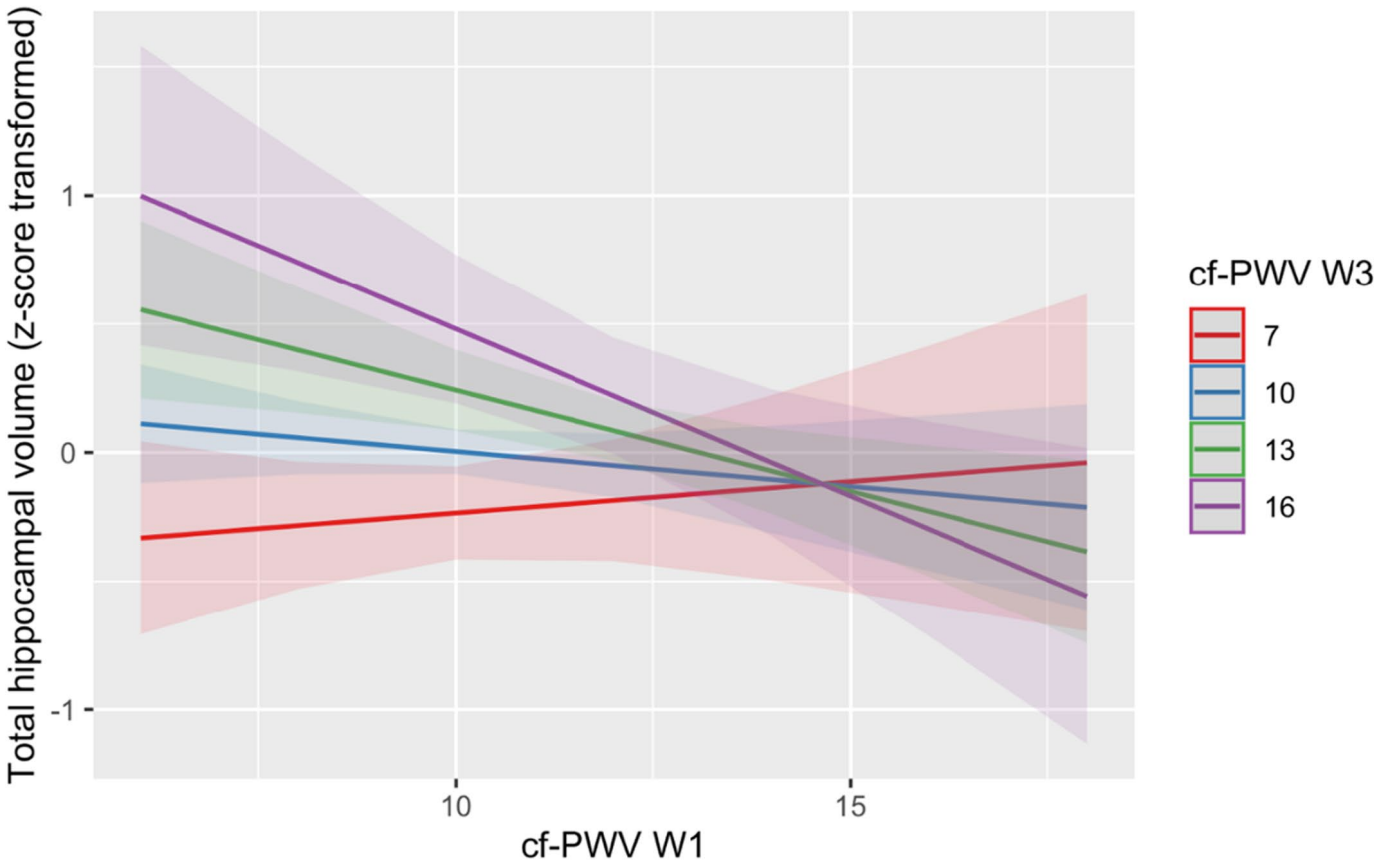
The combined effects of *Cf*-PWV at Wave 1 and cf-PWV at Wave 3 on total hippocampal volume. *Cf*-PWV = Carotid-femoral pulse wave velocity (m/s); W1/3 = Wave 1/3. The interaction between *Cf*-PWV at Wave 1 and *Cf*-PWV at Wave 3 was significant (*p* = 0.03). The combination of high *Cf*-PWV at Wave 1 and high *Cf*-PWV at Wave 3 was associated with total lower hippocampal volume.

When adjusted for *Cf*-PWV at Wave 3 in an additive model, higher *Cf*-PWV at Wave 1 was borderline associated with lower hippocampal volume (B = -0.02; 95% CI = −0.03, 1.64 × 10^−4^; *p* = 0.05). A model including the interaction between *Cf*-PWV at Wave 1 and *Cf*-PWV at Wave 3 demonstrated a significant effect (B = −0.02; 95% = −0.02, −0.002; *p* = 0.03; Table 2, Panel A, Figure 2) such that higher *Cf*-PWV at Wave 1 WITH *Cf*-PWV at Wave 3 was associated with lower total hippocampal volume.

**Figure 3:**
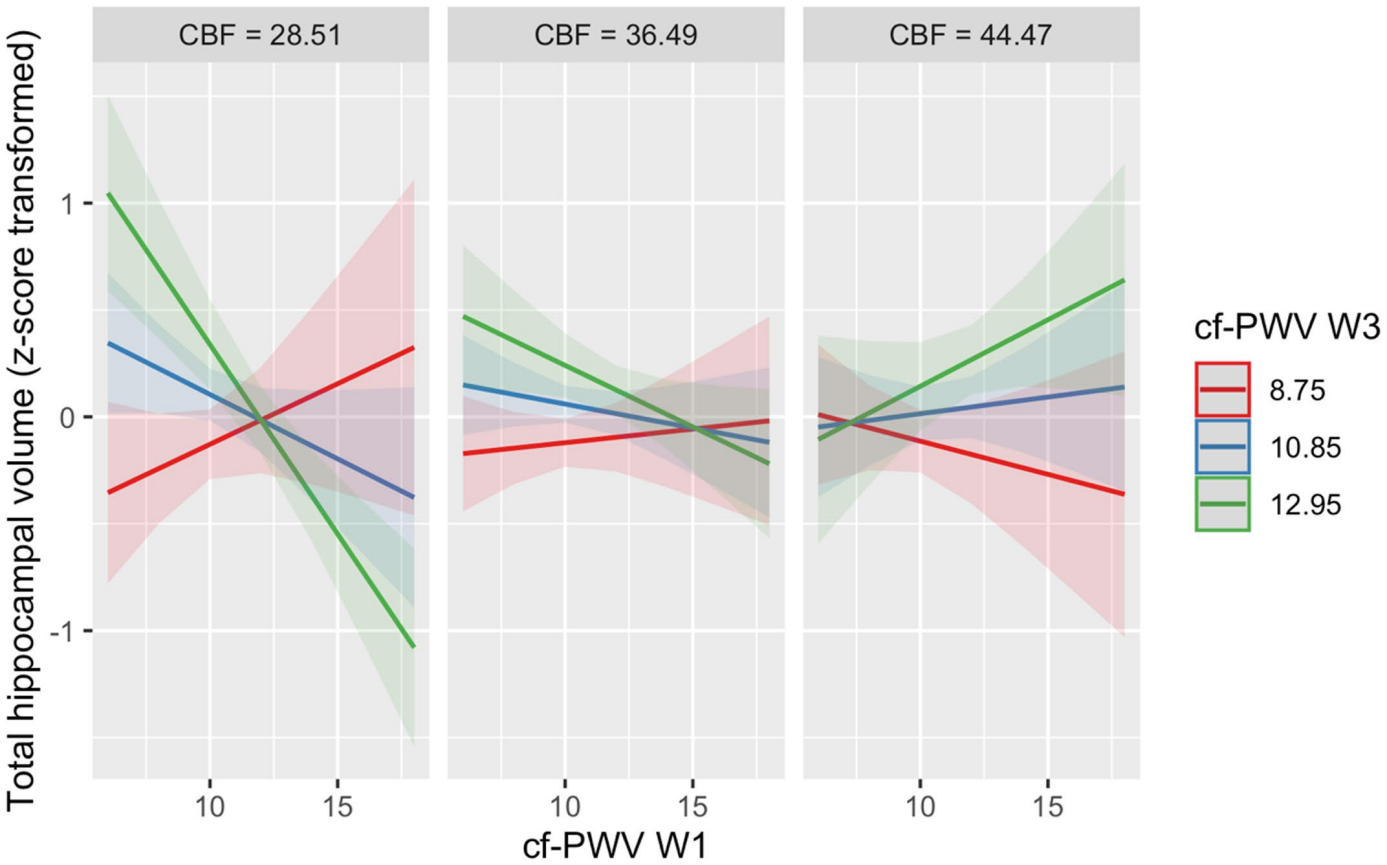
The combined effects of arterial stiffness (Cf-PWV) at Wave 1, arterial stiffness (Cf-PWV) at Wave 3 and CBF at Wave 3 on total hippocampal volume. *Cf*-PWV = Carotid-femoral pulse wave velocity (m/s); W1/3 = Wave 1/3; CBF_GM =_ Mean whole brain grey matter cerebral blood flow (ml/100 g/min). The three-way interaction between *Cf*-PWV at Wave 1, *Cf*-PWV at Wave 3 and CBF_GM_ at Wave 3 was significant (*p* < 0.001). The combination of a 4-year persistent high AS and low CBF_GM_ (high *Cf*-PWV at Wave 1, high *Cf*-PWV at Wave 3 and lower CBF_GM_) is associated with lower hippocampal volume.

The three-way interaction between *Cf*-PWV at Wave 1, *Cf*-PWV at Wave 3 and CBF at Wave 3 shows an effect of a 4-year persistently high AS and low CBF on total hippocampal volume (B = 4.88 × 10–3, 95% CI = 2.63 × 10–3, 7.14 × 10–3; *p* < 0.01), with the interaction between higher *Cf*-PWV at Wave 1, higher *Cf*-PWV at Wave 3 and lower CBF being associated with lower hippocampal volume (Table 2, Panel C, Figure 3). Similar findings were observed in fully adjusted models (Supplementary Table 5).

### Supplementary analyses

#### Cross-sectional

In baseline models, the interaction between cf-PWV and hippocampal subfield volumes at Wave 3 was significant (χ^2^_(15)_ = 10.5; *p* = 0.03). There was a positive relationship between cf-PWV and the volumes of the subfields CA1, CA3 and CA4 (Supplementary Table 6, Panel A/Supplementary Figure 2, Panel A). The interaction between CBF and subfields at Wave 3 was also significant (χ^2^_(15)_ = 9.72; *p* = 0.04). There was a positive relationship between CBF and the volumes of the subiculum and DG (Supplementary Table 6, Panel B/Supplementary Figure 2, Panel B). The associations were robust to mutual adjustment (Supplementary Table 6, Panel C). The interaction between cf-PWV, CBF and subfields at Wave 3 was significant (*p* = 0.01; Supplementary Table 6, Panel D; Supplementary Figure 2, Panel C). Increased AS (high cf-PWV) with preserved CBF at Wave 3 was associated with larger subfield volumes. However, the interaction between high AS and low CBF was not significant, except for the CA3 volume. Similar findings were observed in fully adjusted models (Supplementary Table 7).

#### Follow-up

In baseline models, the interaction between cf-PWV at Wave 1 and hippocampal subfield volumes at Wave 3 was significant (χ^2^_(15)_ = 14.9; *p* < 0.01). There was a negative relationship between cf-PWV at Wave 1 and the volumes of the subiculum, DG and CA4 subfields (Supplementary Table 8, Supplementary Figure 3, Panel A), which were robust to CBF adjustment (Supplementary Table 8, Supplementary Figure 3, Panel B). Only associations with the DG and CA4 remained significant after adjustment for cf-PWV at Wave 3 (Supplementary Table 8, Supplementary Figure 3, Panel C). The interaction between cf-PWV at Wave 1, cf-PWV at Wave 3 and subfields was not significant (*p* = 0.55) nor the interaction between cf-PWV at Wave 1, CBF at Wave 3 and subfields (*p* = 0.33). The interaction between higher cf-PWV at Wave 1 and higher cf-PWV at Wave 3 was associated with lower volumes across subfields (Supplementary Figure 3, Panel B) as was the interaction of higher cf-PWV at Wave 1 and lower CBF at Wave 3 (Supplementary Figure 3, Panel C). Finally, the interaction between cf-PWV at Wave 1, cf-PWV at Wave 3, CBF at Wave 3 and subfields was not significant (*p* = 0.46). The interaction of higher cf-PWV at Wave 1, higher cf-PWV at Wave 3 and lower CBF at Wave 3 was associated with lower volumes across subfields too. Similar findings were observed in fully adjusted models (Supplementary Table 9).

## Discussion

We examined the independent and moderating effects of elevated arterial stiffness and reduced cerebral blood flow on hippocampal volumes and subfield volumes, in a relatively large MRI sample of community-dwelling older adults from ‘The Irish Longitudinal Study on Ageing’.

Our cross-sectional analyses showed that there were no independent associations between raised AS or reduced CBF with total hippocampal volumes. Longitudinal analyses of arterial stiffness suggested that higher AS in the preceding 4 years was independently associated with lower total hippocampal volumes when adjusted for AS at wave 3 in an additive model, however this effect was small. Significant associations between carotid compliance (a surrogate marker for arterial stiffness) and hippocampal volume have been observed in a study of 614 subjects at 20-year follow-up MRI as part of the Atherosclerosis Risk in the Community (ARIC) Study (Baradaran et al., 2021). Carotid compliance was calculated by continuous measurement of the variation in carotid artery diameter during the cardiac cycle, using ultrasound. It is defined as the ‘absolute volume increase within the carotid artery segment during the cardiac cycle, divided by the arterial pulse pressure’. Therefore, the lower the carotid compliance value, the greater the degree of arterial stiffness. Whilst cf-PWV has been consistently cited as the ‘gold standard’ measure of arterial stiffness, conversion tools have been utilised to convert carotid compliance values to local-PWV values, which show significant correlation to the patients cf-PWV measurements (Alhalimi et al., 2021).

Similarly, Bown’s longitudinal study of 278 adults over the age of 60 who had serial MRI brain studies at baseline, 18 months, 3, 5 and 7 years as part of the Vanderbilt Memory and Ageing Project, showed that an elevated baseline pulse wave velocity was associated with a greater decrease in hippocampal volume over time (Bown et al., 2021). As per the SMART-MR Study, the association between cerebral blood flow measurements and hippocampal volumes were lost after adjustment for age and sex (Knoops et al., 2009).

Our analyses examining the combined effects of raised AS and reduced CBF on total hippocampal volume (HV) and the prolonged effect of raised AS over a four-year period showed that (i) prolonged elevated AS (at wave 1 and 3), (ii) the interaction between higher AS at Wave 1 (baseline) and lower CBF at Wave 3 and (iii) the interaction between prolonged elevated AS (at wave 1 and wave 3) and reduced CBF at wave 3 were associated with smaller hippocampal volumes. Our findings suggest a lag effect in the arterial stiffness and hippocampal volume relationship. We propose that the subsequent reduction in cerebral blood flow observed with elevated arterial stiffness may be the missing link in the pathway linking arterial stiffness to hippocampal atrophy. This association between raised arterial stiffness and reduced cerebral blood flow is already reported in the literature in both animal and human subjects (Ajamu et al., 2021; Suri et al., 2020; Sadekova et al., 2018; Jefferson et al., 2018; Muhire et al., 2019). However, contrary to our expectations, reduced CBF at wave 3 in isolation was not associated with hippocampal atrophy, nor was elevated arterial stiffness at Wave 1. Only the combination of both in a model including an interaction term was associated with reduced HV, suggesting CBF plays a buffering effect in the presence of elevated AS. Total hippocampal volume was smaller only in the presence of both higher AS and lower CBF whereas the association between AS and HV was positive in the presence of preserved CBF. The literature does not fully explain why this may be the case. Perhaps when arterial stiffness starts to increase, there is an initial compensatory mechanism that transiently increases flow, but with prolonged exposure, this relationship reverses. A number of authors have proposed that this is due to the fact that in healthy individuals, a compensatory reduction in vascular bed resistance occurs in response to cerebral hypoperfusion, and this maintains adequate cerebral perfusion (Marstrand et al., 2002; Mitchell et al., 2011). Perhaps in the setting of progressive cerebral small vessel disease (i.e increased arterial stiffness), this autoregulation is impaired and thus increased AS leads to an increase in the transmission of harmful pulsatility into the thin-walled cerebral microcirculation, which is unequipped to deal with this increased pressure. This could, over time, ultimately result in vascular remodelling, and resultant reduction in cerebral blood flow and perfusion (Marstrand et al., 2002; Mitchell et al., 2011; Mitchell, 2021). The structure of the hippocampus makes it especially vulnerable to ischemia. The blood supply to the hippocampus is derived primarily from branches of the posterior cerebral artery (PCA) and contributory vessels from the anterior choroidal artery. Animal studies have examined the microvasculature of the hippocampus and unanimously report that the hippocampus has one of the lowest microvascular densities, relative to the surrounding cerebral cortices (Shaw et al., 2021; Nair et al., 1960; Zhang et al., 2019; Kirst et al., 2020).

The hippocampus also has a relatively long distance between its micro vessels and parent supplying artery. On top of this, the hippocampus is highly sensitive to hypoxia and ischemic insult with its high metabolic demand, and this therefore creates a perfect storm scenario whereby the hippocampus is highly vulnerable to vascular insult whilst simultaneously being ill-equipped to deal with states of hypoperfusion (Muhire et al., 2019; Wang and Michaelis, 2010). Therefore, maintaining adequate hippocampal perfusion is essential to preserve cognitive and memory function.

We hypothesised that the CA-1 subfield of the hippocampus would demonstrate preferential vulnerability to ischemia, compared to the other subfields examined; CA2, CA3, CA4, and DG. We hypothesized from our literature search that the CA4 and subiculum may also show regional vulnerability to raised arterial stiffness. However, this hypothesis was only partially confirmed by our results. The supplementary analyses demonstrated that the subiculum and the DG may in fact be most sensitive to higher AS and lower CBF, but these results were not consistent across our analyses. Also, a positive cross-sectional association was observed between AS and the CA-1, CA-3 and CA-4 subfields. The analyses examining these interaction effects suggest a similar effect of raised AS and reduced CBF across all subfields, with smaller subfield volumes observed across the board, as per total HV.

Whilst no prior study, to the best of our knowledge, has specifically examined the effects of increased arterial stiffness and reduced cerebral blood flow on hippocampal subfield volumes, several authors have examined the link between vascular risk factors on specific hippocampal subfield volumes as well as animal models which have investigated the effects of global brain ischemia on the hippocampus and its subfields (Wilde et al., 1997; Sugawara et al., 2000; Wu et al., 2008; Shing et al., 2011).

CA-1 is widely reported in the literature (Sugawara et al., 2000; Wu et al., 2008; Shing et al., 2011) to be the subfield that harbours the most sensitivity to ischemia and hypoxia and thus we tested the independent and moderating effects of AS and CBF on the subfields CA3, CA4, DG and subiculum, with the CA-1 as a reference level. However, this effect of hippocampal atrophy with raised AS and reduced CBF, contrary to our hypothesis, was exerted equally across all subfields tested. We had hypothesised that the CA-1 subfield would be most vulnerable owing to the vascularisation of the hippocampus and the fragility of its vascular network (Spallazzi et al., 2019; Duvernoy et al., 2013; Marinkovic et al., 1999), but this was not borne out in our study. However, along with several authors we observed small independent associations between elevated AS and reduced CBF with smaller volumes of the subiculum (Kim et al., 2015; Pin et al., 2021; Wu et al., 2008; Li et al., 2016).

Pin et al. investigated the effects of neurovascular damage on hippocampal subfields in their study of 249 non-demented adults who underwent two 1.5 Tesla MRI scans, 4 years apart. They examined the association between the atrophy of the CA1-3, CA4 and subiculum with the presence of white matter hyperintensities (WMH’s), which are thought to result from chronic hypoxia (Pin et al., 2021; Tubi et al., 2020). Interestingly, they reported that the subiculum was the only tested subfield in which volume loss correlated with white matter hyperintensities, independent of other vascular risk factors. Whilst these authors did not examine the impact of raised arterial stiffness or reduced cerebral blood flow on hippocampal subfield volumes, they used white matter hyperintensities as a marker of cerebral small vessel disease and linked the increasing quantities of WMH’s to the increasing rates of subiculum atrophy in their longitudinal analyses of their study cohort (Pin et al., 2021). The findings from our study of healthy ageing adults supports the findings of Pin (Pin et al., 2021) and other authors (Kim et al., 2015; Li et al., 2016) and highlight the subiculum as a hippocampal subfield of interest that may demonstrate preferential sensitivity to hypoxia, ischemia, and cerebrovascular insult. There is an obvious paucity of recent research in the literature examining this selective vulnerability of specific subfields to ischemia and in particular the effects of a combination of raised arterial stiffness and reduced cerebral blood flow on the subfield volumes. The hippocampus is the principal site of neurogenesis (specifically within the dentate gryus) and the rate of hippocampal neurogenesis has been shown to decline in patients with Alzheimer’s dementia compared to healthy subjects (Moreno-Jimenez et al., 2019; Lempriere, 2019). This is important in the identification of possible modifiable risk factors that can be used to treat patients with hippocampal vascular insults and thus impaired neurogenesis. Medications such as angiotensin-converting enzymes inhibitors, angiotensin II receptor blockers and calcium channel blockers have been demonstrated to reduce arterial stiffness, promote vascular remodelling and improve endothelial function (Dudenbostel and Glasser, 2012). Further research is required to establish whether these benefits may be extended to the improvement of hippocampal perfusion and neurogenesis.

It is important to better understand this long-accepted link between vascular disease and cognitive decline in order to develop potential therapeutic targets. Better understanding of the pattern of hippocampal atrophy within distinct subfields will help clinicians identify patients that are at risk of cognitive decline. Thus, by mitigating a patient’s vascular risk profile we may reduce their risk of embarking on this pathway of elevated arterial stiffness, reduced cerebral blood flow, subsequent hippocampal atrophy and utimately cognitive decline.

### Limitations

Firstly, loss to follow-up patients and missing data in others meant that the study sample was overall healthier than those excluded; therefore, the generalizability of our findings is less certain, but we believe this does not negatively impact upon the observed associations.

Second, although PWV was examined in a follow-up manner (measurement repeated at 4-year follow up), there were no repeated measures for cerebral blood flow and hippocampal volumes and thus a causal link between arterial stiffness, cerebral blood flow and hippocampal volumes cannot be established. Our follow-up analyses suggest that prolonged exposure to elevated arterial stiffness may over time have an effect on hippocampal volume. Further research examining changes in AS, CBF and HV over time is warranted to fully understand these relationships.

Third, we used a measurement of total cerebral blood flow. However, hippocampal blood flow measurements may help to better understand the effect of CBF on HV. It is possible that the null independent relationship observed between CBF and HV was linked to a lack of specificity (total CBF versus hippocampal blood flow specifically).

Fourth, there are some limitations to automatic segmentation of the hippocampal subfields such that the segmentation may be based off low-resolution T1-weighted images. Difficulty in distinguishing some specific subfields such as the molecular layer have been reported in the literature (Iglesias et al., 2015; Wisse et al., 2021) and a combination of T1 and T2 weighted images for the purpose of segmentation has been proposed. A combined T1 and T2 weighted approach for the purpose of subfield segmentation was not possible in our study due to the varying in-plane resolutions between the two (T1w images were 0.9 mm isotropic; T2 weighted images were 0.58 × 0.72 × 4 mm). The alternative approach, validation of automated FreeSurfer segmentation with manual segmentation would be a time-costly process, with an estimated 50 h of manual segmentation required per case (Iglesias et al., 2015). This impracticality would limit the sample size possible for such analyses. Our relatively large sample size and the consistency of our methodology with respect to other large cohort studies (i.e UK Biobank) afford the opportunity for our results to be replicated in future studies.

Finally, this study focused on a specific subset of hippocampal subfield volumes that had been previously identified from the literature search. This was performed in order to achieve sufficient power for the statistical analysis.

### Strengths

To our knowledge, this is the first study to examine the independent and moderating effects of elevated arterial stiffness and reduced cerebral blood flow on hippocampal volumes and subfield volumes in a relatively large MRI sample of community-dwelling older adults from a population-based study. Our analyses were also adjusted for a large range of confounding factors including age, sex, education status, cardiovascular comorbidities, medications, and lifestyle factors. It is also the first study to explore the effect of prolonged exposure of elevated AS over a four-year follow up period on hippocampal subfield volumes, the closest study coming from Pin et al. who looked at hippocampal subfield volume loss and WMH’s over a 4 year follow-up period. Other studies looking at specific subfields have been smaller and cross-sectional in nature (Kim et al., 2015; Li et al., 2016), and have not specifically examined the independent and combined effects of raised AS and reduced CBF on the hippocampus. While this is the first attempt to disentangle these effects on the hippocampus, this study should however be considered preliminary and exploratory but supports the need for future work examining changes in AS, CBF and HV over a longer period of time.

## Conclusion

Our study shows that increased arterial stiffness and reduced CBF are not independently associated with whole hippocampal or hippocampal subfield volumes. However, when combined, increased arterial stiffness for a longer duration (spanning the 4 year follow-up period) in combination with a reduction in cerebral blood flow is associated with lower hippocampal volumes. These effects were equally exerted across all hippocampal subfields tested in this study.

Improving our knowledge surrounding the mechanisms that lead to the hippocampal atrophy has clinical implications. Understanding the hippocampal vasculature and its response to hypoperfusion is critical in the development of potential therapeutic targets in patients with an elevated vascular risk profile. Targeting these vascular risk factors, such as arterial stiffness, may help lower the rate of hippocampal atrophy and thus slow the onset of cognitive impairment in our ageing population.

## Data Availability

The raw data supporting the conclusions of this article will be made available by the authors, without undue reservation.
