# Supplementary material for: Examining the independent and moderating effects of arterial stiffness and cerebral blood flow on total hippocampal and hippocampal subfield volumes
Source: Front Aging Neurosci. 2025 Jun 30;17:1466294. doi: 10.3389/fnagi.2025.1466294 (PMC12264913; doi:10.3389/fnagi.2025.1466294)
Supplement: Supplementary file 1 [file Data_Sheet_1.docx]

**Supplementary Figure 1:** Flowchart detailing patient selection and enrolment in study. N=395 final number of patients enrolled.

Wave 3 participants aged 50+

N=6687

Home-based health assessment (1082)

or did not attend HA (1296)

N=2378

Center-based health assessment

N=4309

Did not undergo brain MRI

N=3731

MRI sub-study

N=578

No data obtained due to claustrophobia/ nervousness (14) or MRI contraindication (4)

N=18

MRI data acquired

N=560

No reliable PWV data at Wave 1 and Wave 3 and CBF data

N=69

MRI data available for analysis

N=464

**Analysis sample**

**N=395**

Data quality issues (71), lesions (18) & TIA (5) & stroke (2)

N=96

*Note: HA: Health assessment; MRI: Magnetic resonance imaging.*

**Supplementary Figure 2, Panel A.** Relationship between PWV-W3 and subfield volumes.


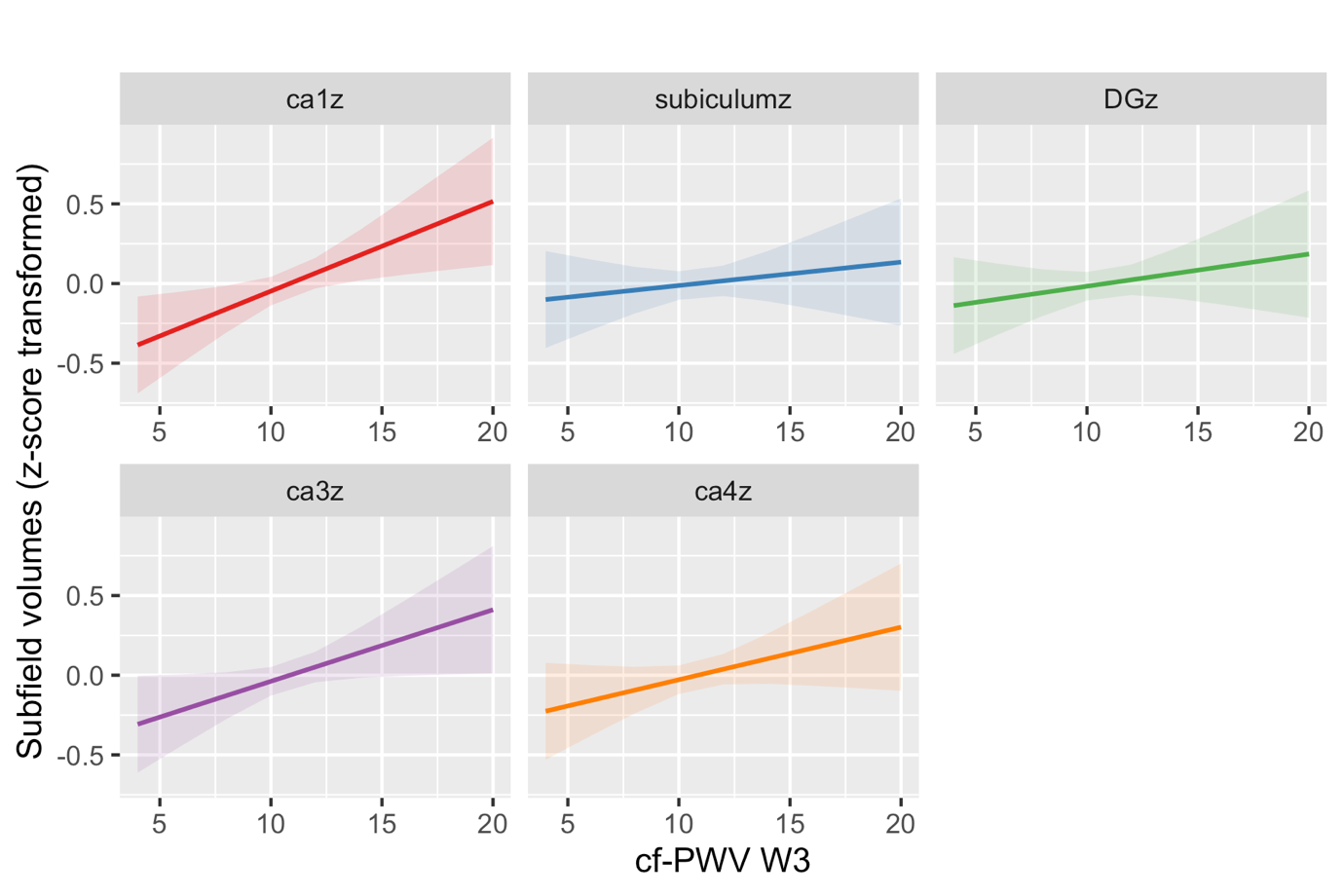


*Note:* Cf-PWV = Carotid-femoral pulse wave velocity (m/s) ; W1/3 = Wave 1/3. There was a positive relationship between cf-PWV at wave 3 and the volumes of the subfields CA1, CA3 and CA4.

**Supplementary Figure 2, Panel B.** Relationship between CBF and subfield volumes.


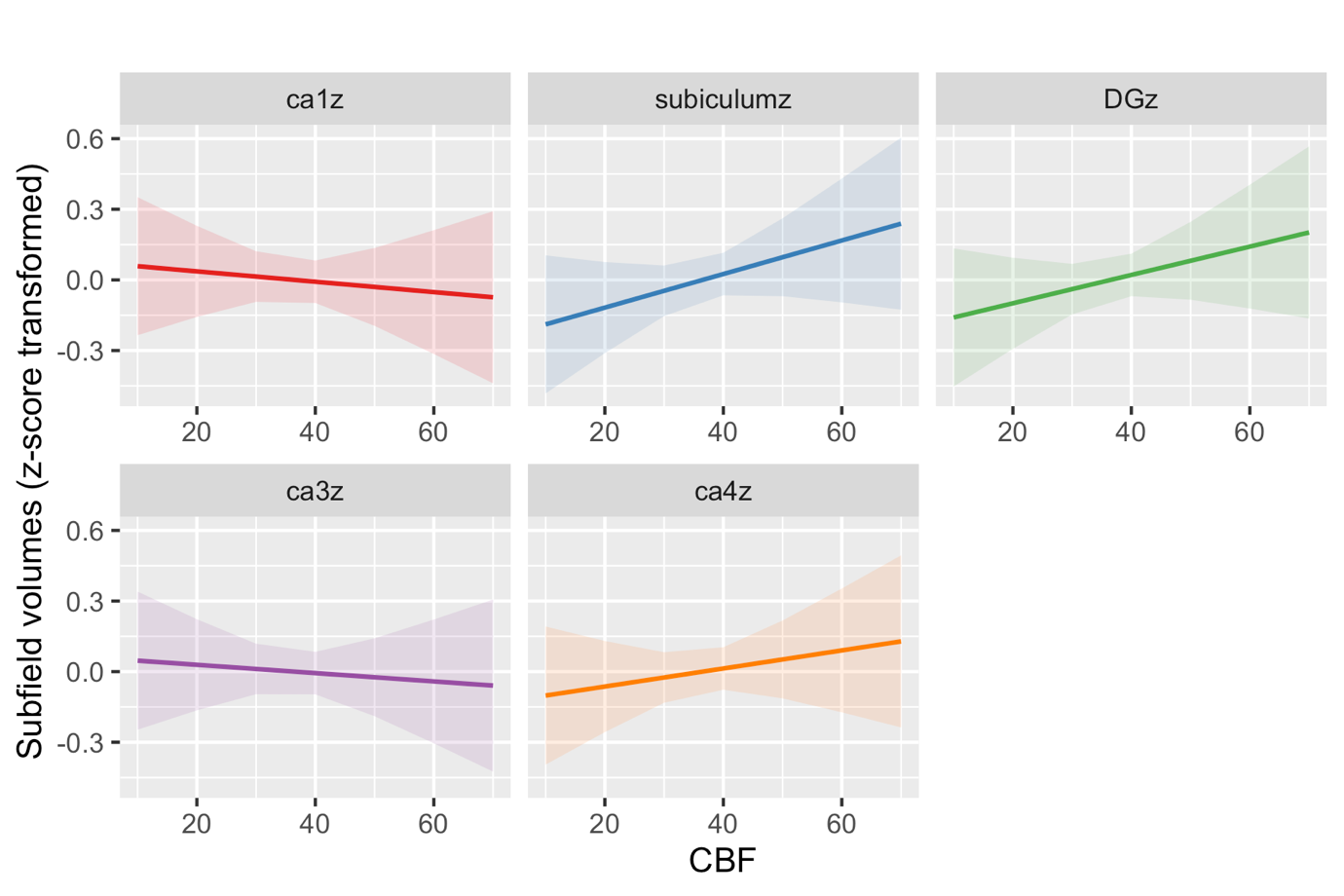


*Note:* W1/3 = Wave 1/3; CBF_GM =_ Mean whole brain grey matter cerebral blood flow (ml/100g/min). There was a positive relationship between CBF and the volumes of the subiculum and DG.

**Supplementary Figure 2, Panel C.** Interaction between PWV-W3, CBF and subfield volumes


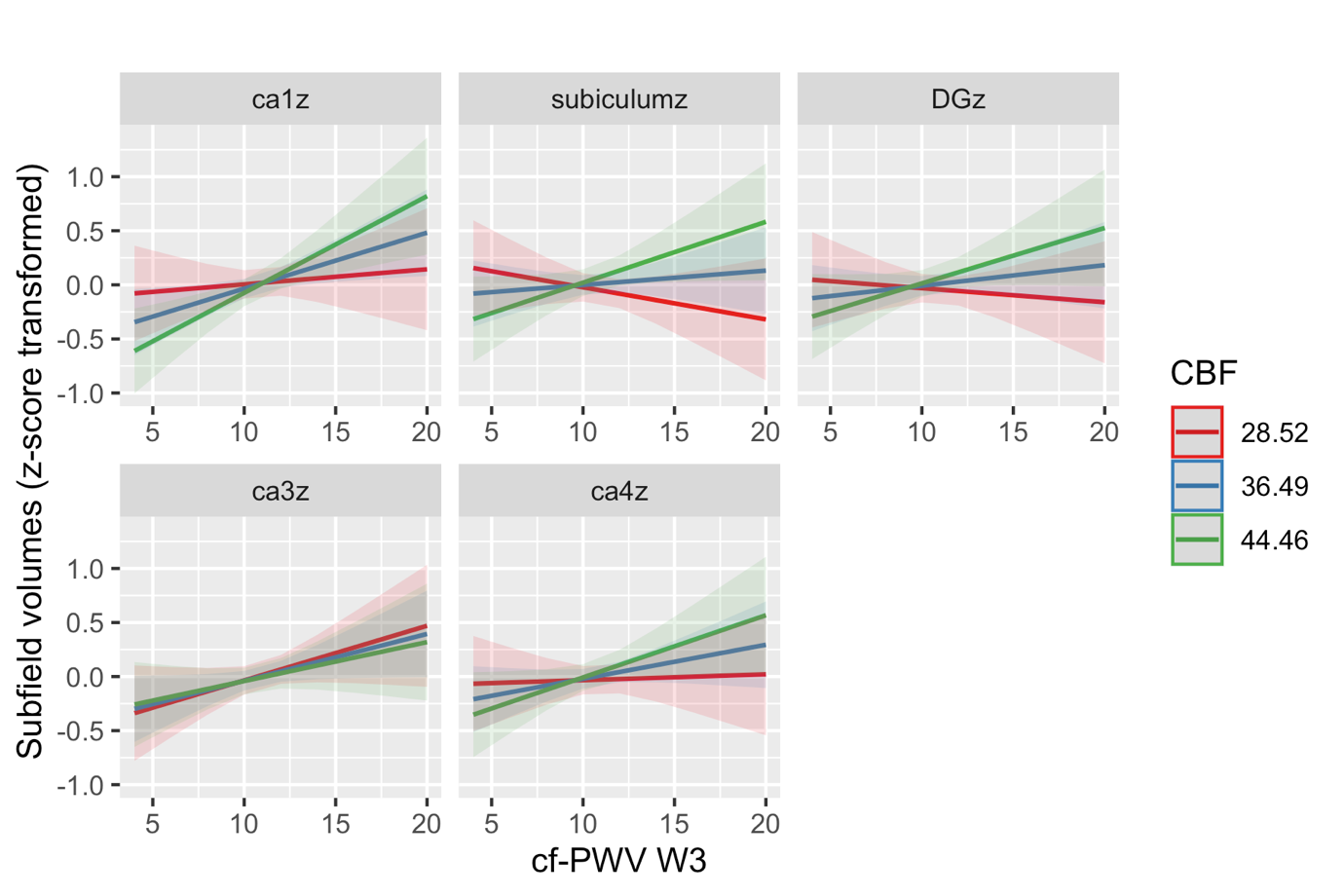
*Note:* Cf-PWV = Carotid-femoral pulse wave velocity (m/s); W1/3 = Wave 1/3; CBF_GM =_ Mean whole brain grey matter cerebral blood flow (ml/100g/min). The interaction between cf-PWV, CBF and subfields at Wave 3 was significant (p=0.01). The combination of high AS and high CBF at Wave 3 was associated with larger subfield volumes while the co-occurrence of higher AS and lower CBF was not, except for the CA3 volume.

**Supplementary Figure 3, Panel A.** Relationship between cf-PWV at wave 1 and the subfield volumes. **
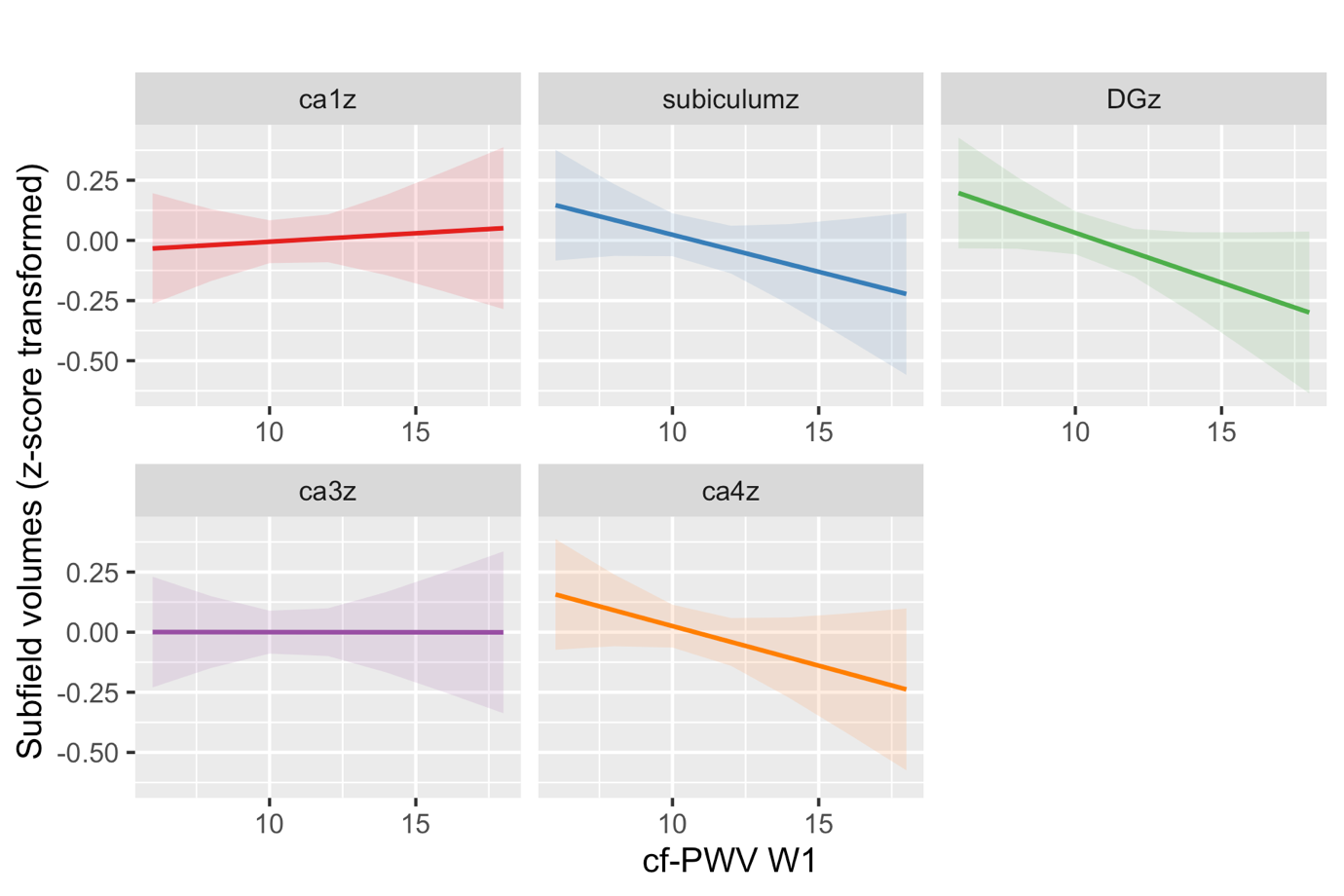
**

*Note:* Cf-PWV = Carotid-femoral pulse wave velocity (m/s); W1/3 = Wave 1/3. There was a negative relationship between cf-PWV at Wave 1 and the volumes of the subiculum, DG and CA4 subfields.

**Supplementary Figure 3, Panel B.** The interaction between cf-PWV at Wave 1, cf-PWV at Wave 3 and subfield volumes.

**
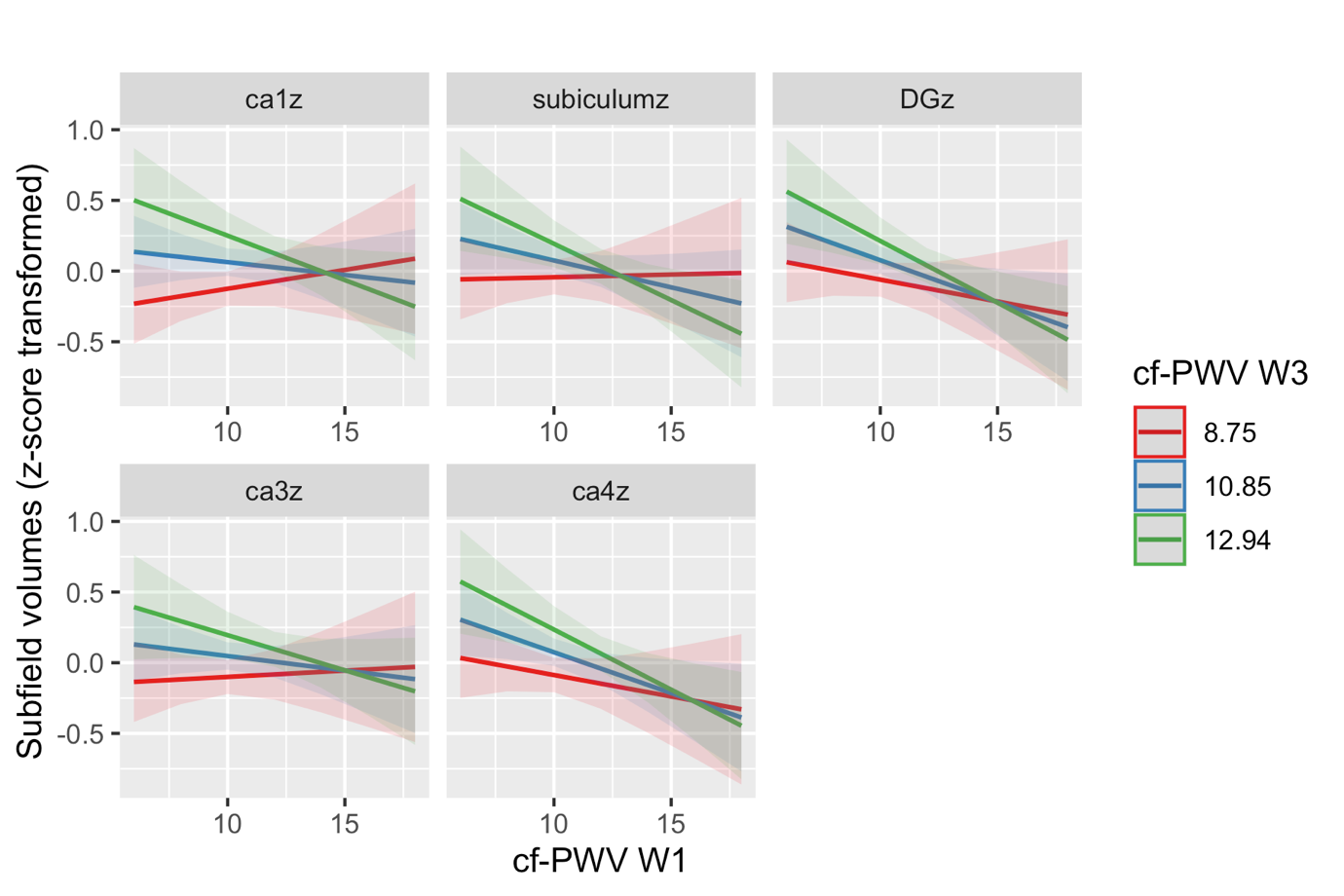
**

*Note:* Cf-PWV = Carotid-femoral pulse wave velocity (m/s) ; W1/3 = Wave 1/3. The interaction between cf-PWV at Wave 1, cf-PWV at Wave 3 and subfield volumes was not significant. The interaction between high cf-PWV at Wave 1 and high cf-PWV at Wave 3 was associated with lower volumes across all subfields.

**Supplementary Figure 3, Panel C.** The interaction between cf-PWV at wave 1 and CBF_GM_ at Wave 3.

*
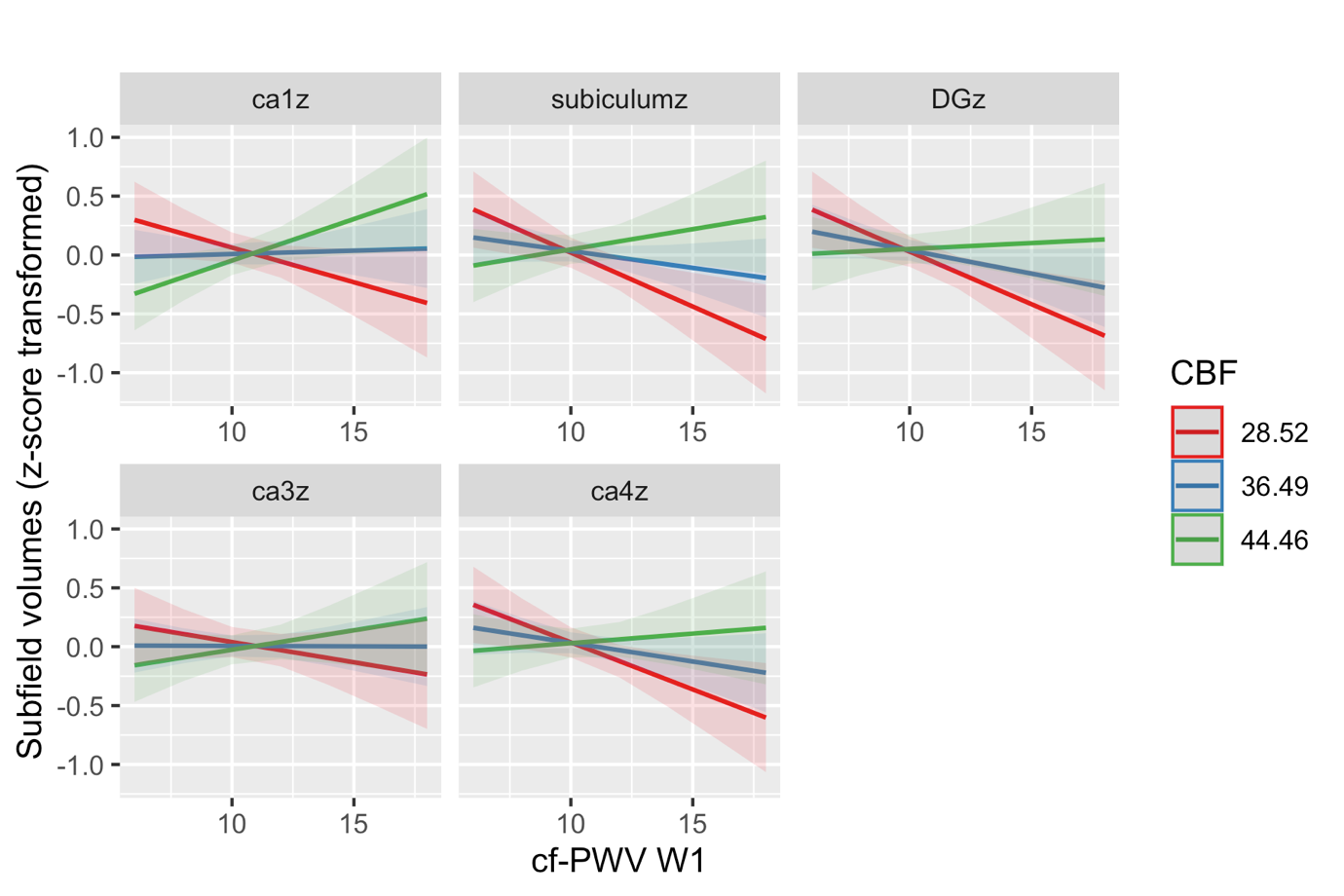
*

*Note:* Cf-PWV = Carotid-femoral pulse wave velocity (m/s); W1/3 = Wave 1/3; CBF_GM =_ Mean whole brain grey matter cerebral blood flow (ml/100g/min). The interaction between high cf-PWV at Wave 1 and low CBF_GM_ at Wave 3 was associated with lower volumes across all subfields.

**Supplementary Table 1.** Sample characteristics of participants with and without evidence of arterial stiffness (cf-PWV <12m/s and >12m/s) at Wave 1.

|  | Cf-PWV W1 (<=12) (N=345; 87%) | Cf-PWV W1 (> 12) (N=50) (13%) |
| --- | --- | --- |
| Age (mean, sd) years | 67.5 (6.8) | 73.3 (7.4)^***^ |
| Sex (Female, %) | 54.4 | 52.0 |
| Education (Low, %) | 20.0 | 22.0 |
| CVD conditions (>1, %) | 60.3 | 70.0 |
| Systolic/ Diastolic BP (mean, sd) mmHg | 132.5 (18.5)/ 79.5 (10.1) | 143.8 (18.6) ^***^/ 82.3 (10.4) |
| Antihypertensives (on meds, %) | 14.4 | 16.0 |
| BMI (mean, sd) | 27.9 (4.3) | 27.7 (4.1) |
| Smoking (Present, %) | 5.5 | 4.0 |
| Physical activity (low, %) | 35.6 | 31.2 |
| Problematic alcohol (%) | 9.7 | 10.8 |
| Cf-PWV at W3 m/s | 10.5 (1.8) | 12.9 (2.4) ^***^ |
| CBF_GM_ ml/100g/ml | 36.5 (7.9) | 36.2 (8.1) |

*Note: t*-tests and chi-squared tests (where appropriate) were used to assess differences between the two groups.*** (p<0.001), ** (p<.01) and * (*p* < .05) indicate significant differences. Cf-PWV = Carotid-femoral pulse wave velocity; W1/3 = Wave 1/3; CBF_GM =_ Mean whole brain grey matter cerebral blood flow.

**Supplementary Table 2.** Sample characteristics of participants with and without evidence of arterial stiffness (cf-PWV <12m/s and >12m/s) at Wave 3.

|  | Cf-PWV W3 (<=12) (N=337; 85%) | Cf-PWV W3 (> 12) (N=58) (15%) |
| --- | --- | --- |
| Age (mean, sd) years | 67.5 (6.8) | 72.8 (7.4) ^***^ |
| Sex (Female, %) | 55.4 | 46.5 |
| Education (Low, %) | 20.7 | 17.2 |
| CVD conditions (>1, %) | 62.6 | 55.1 |
| Systolic/ Diastolic BP (mean, sd) | 132.4 (18.5)/ 79.5 (9.6) | 142.6^***^ (18.7)/ 81.5 (12.8) |
| Antihypertensives (on meds, %) | 14.8 | 13.7 |
| BMI (mean, sd) kg/m^2^ | 27.9 (4.3) | 27.3 (4.2) |
| Smoking (Present, %) | 5.0 | 6.8 |
| Physical activity (low, %) | 35.9 | 30.3 |
| Problematic alcohol (%) | 9.9 | 10.0 |
| PWV at W1 m/s | 10.5 (1.8) | 12.3 (2.1) ^***^ |
| CBF ml/100g/min | 36.6 (8.0) | 35.5 (7.4) |

*Note: t*-tests and chi-squared tests (where appropriate) were used to assess differences between the two groups.*** (p<0.001), ** (p<.01) and * (*p* < .05) indicate significant differences. Cf-PWV = Carotid-femoral pulse wave velocity; W1/3 = Wave 1/3; CBF_GM =_ Mean whole brain grey matter cerebral blood flow.

**Supplementary Table 3.** Sample characteristics of participants per whole brain grey matter cerebral blood flow (CBF_GM_) tertile at Wave 3.

|  | CBF Tertile 1 (n=, %) | CBF Tertile 2 (n,%) | CBF Tertile 3 (n,%) |
| --- | --- | --- | --- |
| CBF_GM_ (mean, sd) ml/100g/min | 28.0 (3.9) | 36.3 (2.1) | 45.2 (4.9) |
| Age (mean, sd) years | 69.6 (7.1) | 68.3 (7.5) | 66.92 (6.7)** |
| Sex (Female, %) | 42.4 | 55.3 | 64.9*** |
| Education (Low, %) | 21.97 | 20.45 | 18.32 |
| CVD conditions (>1, %) | 66.67 | 59.1 | 58.78 |
| Systolic/ Diastolic BP (mean, sd) mmHg | 137.85 (19.02) / 82.02 (10.04 | 135.61 (19.85) / 79.87 (10.81) | 128.36 (16.57)*** / 77.58 (9.35)*** |
| Antihypertensives (on meds, %) | 18.18 | 15.15 | 10.69 |
| BMI (mean, sd) | 28.71 (4.23) | 27.84 (4.2) | 27.15 (4.29)** |
| Smoking (Present, %) | 3.03 | 8.33 | 4.58 |
| Physical activity (low, %) | 35.25 | 34.11 | 35.94 |
| Problematic alcohol (%) | 11.76 | 11.3 | 6.7 |
| PWV at W1 (mean, sd) m/s | 11.04 (1.9) | 10.68 (2.07) | 10.57 (1.95) |
| PWV at W3 (mean, sd) m/s | 11.14 (2.06) | 10.91 (2.02) | 10.48 (2.18)** |

*Note:* Ordinal logistic regressions were used to assess differences between CBF tertiles, with CBF Tertile 1 taken as the reference category.*** (p<0.001), ** (p<.01) and * (*p* < .05) indicate significant differences. Cf-PWV = Carotid-femoral pulse wave velocity; W1/3 = Wave 1/3; CBF_GM =_ Mean whole brain grey matter cerebral blood flow.

**Supplementary Table 4.** Estimates and 95% confidence intervals of the effects of arterial stiffness (Cf-PWV) at Wave 3 and CBF at Wave 3 on total hippocampal volume from fully adjusted models that also control for education, cardiovascular disease and events, blood pressure, anti-hypertensives and lifestyle factors..

|  | Panel A: Independent effect of AS on hippo volume | | Panel B: Independent effect of CBF on hippo volume | | Panel C: Combined effects of AS and CBF on hippo volume | |
| --- | --- | --- | --- | --- | --- | --- |
|  | Estimates (95% CI) | p-value | Estimates (95% CI) | p-value | Estimates (95% CI) | p-value |
| PWV-W3 | 0.04 (-0.01, 0.07) | 0.06 | - | - | -0.10 (-0.28,0.08) | 0.27 |
| CBF | - | - | 0.01 (-0.002, 0.02) | 0.13 | -0.03 (-0.08, 0.02) | 0.23 |
| PWV x CBF | - | - | - | - | 0.004 (-0.001,0.01) | 0.13 |
| Covariates | | | | | | |
| Age | -0.08 (-0.09,-0.066)( | *** | -0.07 (-0.086, -0.06) | *** | -0.08 (-0.09, -0.06) | *** |
| Sex | -0.31 (-0.51, -0.099) | ** | -0.33 (-0.54,-0.13) | ** | -0.32 (-0.52, -0.11) | ** |
| eTIV | 0.37 (0.27, 0.47) | *** | 0.37 (0.27, 0.47) | *** | 0.38 (0.28, 0.48) | *** |
| Education | -0.07 (-0.18, 0.04) | 0.19 | -0.064 (-0.17, 0.04) | 0.23 | -0.06 (-0.17,0.04) | 0.22 |
| CVD conditions | -0.02 (-0.19, 0.14) | 0.78 | -0.04 (-0.21, 0.13) | 0.65 | -0.02 (-0.19, 0.15) | 0.81 |
| Systolic BP | 0.01 (0.001, 0.014) | * | 0.01 (0.002, 0.015) | ** | 0.01 (0.001, 0.1) | * |
| Diastolic BP | -0.01 (-0.02, 0.001) | 0.07 | -0.01 (-0.02, 0.002) | 0.09 | -0.01 (-0.02,0.01) | 0.08 |
| Antihypertensives | 0.13 (-0.095, 0.366) | 0.24 | 0.14 (-0.09, 0.37) | 0.24 | 0.13 (-0.11, 0.36) | 0.28 |
| Smoking | -0.095 (-0.45, 0.26) | 0.6 | -0.09 (-0.45, 0.27) | 0.62 | -0.095 (-0.45, 0.26) | 0.6 |
| Exercise (High level) | -0.13 (-0.34, 0.07) | 0.2 | -0.13 (-0.34, 0.07) | 0.2 | -0.15 (-0.35, 0.06) | 0.15 |
| Alcohol use | -0.061 (-0.28, 0.28) | 0.98 | -0.008 (-0.29, 0.27) | 0.96 | 0.004 (-0.28, 0.24) | 0.98 |
| BMI | -0.011 (-0.03, 0.008) | 0.24 | -0.01 (-0.03, 0.01) | 0.37 | -0.01 (-0.02, 0.01) | 0.37 |

.*** (p<0.001), ** (p<.01) and * (*p* < .05) indicate significant differences.

**Supplementary Table 5.** Estimates and 95% confidence intervals of the independent and additive effects of arterial stiffness (Cf-PWV) at Wave 1, arterial stiffness at Wave 3 and CBF at Wave 3 on total hippocampal volume, in fully adjusted models that also control for education, cardiovascular disease and events, blood pressure, anti-hypertensives and lifestyle factors.

|  | Panel A: Independent effect of AS-wave 1 on hippo volume | | Panel B: Combined effects of AS-Wave 1 and AS-wave 3 on hippo volume | | Panel C: Combined effects of AS-wave 1 and CBF-wave 3 on hippo volume | |
| --- | --- | --- | --- | --- | --- | --- |
|  | Estimates (95% CI) | p-value | Estimates (95% CI) | p-value | Estimates (95% CI) | p-value |
| PWV-W1 | -0.03 (-0.07, 0.02) | 0.22 | 0.09 (-0.06, 0.31) | 0.18 | -0.31 (-0.5, -0.11) | ** |
| PWV-W3 | - | - | 0.23 (0.04, 0.42) | * | - | - |
| CBF-W3 | - | - | - | - | -0.07 (-0.13, -0.018) | ** |
| PWV-W1 x PWV-W3 | - | - | -0.02 (-0.03, 0.0002) | 0.052 | - | - |
| PWV-W1 x CBF | - | - |  | - | 0.008 (0.003, 0.013) | 0.004** |
| Covariates | | | | | | |
| Age | -0.07 (-0.08, -0.06) | *** | -0.08 (-0.09, -0.06) | *** | -0.07 (-0.08, -0.06) | *** |
| Sex | -0.34 (-0.55, -0.14) | ** | -0.3 (-0.5, -0.08) | ** | -0.33 (-0.53, -0.12) | 0.002** |
| eTIV | 0.36 (-0.26, 0.46) | *** | 0.38 (0.28, 0.47) | *** | 0.38 (0.28, 0.48) | *** |
| Education | -0.07 (-0.18, 0.03) | 0.19 | -0.08 (-0.19, 0.02) | 0.13 | -0.07 (-0.18, 0.03) | 0.17 |
| CVD conditions | -0.04 (-0.2, 0.13) | 0.68 | -0.02 (-0.19-0.14) | 0.78 | -0.04 (-0.2, 0.13) | 0.66 |
| Systolic BP | 0.009 (0.003, 0.02) | ** | 0.01 (0.001, 0.014) | * | 0.01 (0.003, 0.02) | 0.1 |
| Diastolic BP | -0.01 (-0.02, 0.001) | 0.07 | -0.01 (-0.02, 0.001) | 0.08 | -0.01 (-0.02, 0.001) | 0.1 |
| Antihypertensives | 0.12 (-0.11, 0.35) | 0.3 | 0.14 (-0.09, 0.36) | 0.25 | 0.11 (-0.12, 0.34) | 0.36 |
| Smoking | -0.06 (-0.42, 0.3) | 0.75 | -0.09 (-0.44, 0,27) | 0.63 | -0.4 (-0.4, 0.3) | 0.84 |
| Exercise (High level) | -0.12 (-0.33, 0.08)) | 0.25 | -0.12 (-0.32, 0.09) | 0.26 | -0.13 (-0.34, 0.07) | 0.2 |
| Alcohol use | -0.02 (-0.3, 0.26) | 0.89 | -0.03 (-0.28, 0.28) | 0.99 | -0.01 (-0.29, 0.27) | 0.96 |
| BMI | -0.01 (-0.03, 0.01) | 0.26 | -0.01 (-0.03, 0.008) | 0.24 | -0.01 (-0.03, 0.01) | 0.37 |

.*** (p<0.001), ** (p<.01) and * (*p* < .05) indicate significant differences.

**Supplementary Table 6:** Baseline model estimates and 95% confidence intervals of the relationship between arterial stiffness (Cf-PWV) at Wave 3 and hippocampal subfield volumes (Panel A), between CBF and subfield volumes (Panel B), these two relationships mutually adjusted (Panel C) and the interaction between PWV-W3, CBF and the hippocampal subfield volumes (Panel D).

|  | Panel A: Relationship between PWV-W3 and subfield volumes | | Panel B: Relationship between CBF and subfield volumes | | Panel C: These relationships (PWV and subfield volumes; CBF and subfield volumes), mutually adjusted | | Panel D: Interaction between PWV-W3, CBF and subfield volumes | |
| --- | --- | --- | --- | --- | --- | --- | --- | --- |
|  | Estimates (95% CI) | p-value | Estimates (95% CI) | p-value | Estimates (95% CI) | p-value | Estimates (95% CI) | p-value |
| PWV-W3 | 0.056 (0.01, 0.1) | ** | - | - | 0.055 (0.01, 0.097) | * | -0.12 (-0.31, 0.07) | 0.22 |
| CBF | - | - | -0.002 (-0.01, 0.008) | 0.68 | -0.001 (-0.01, 0.009) | 0.81 | -0.052 (-0.11, 0.003) | 0.06 |
| PWV-W3 x subiculum | -0.04 (-0.07, -0.012) | ** | - | - | -0.038 (-0.068, -0.009) | * | 0.0007 (-0.003, 0.004) | 0.73 |
| PWV-W3 x DG | -0.036 (-0.07, -0.007) | * | - | - | -0.03 (-0.06, -0.004) | * | -0.0007 (-0.004, 0.003) | 0.71 |
| PWV-W3 x CA-3 | -0.011 (-0.04, 0.018) | 0.45 | - | - | -0.011 (-0.04, 0.02) | 0.45 | -0.006 (-0.009, -0.002) | 0.003** |
| PWV-W3 x CA-4 | -0.023 (-0.05, 0.006) | 0.12 | - | - | -0.021 (-0.05, 0.008) | 0.16 | -0.001 (-0.005, 0.002) | 0.44 |
| CBF x subiculum | - | - | 0.009 (0.002, 0.017) | * | 0.008 (0.0005, 0.016) | * | - | - |
| CBF x DG | - | - | 0.008 (0.0005, 0.016) | * | 0.007 (-0.0005, 0.014) | 0.07 | - | - |
| CBF x CA-3 | - | - | 0.0004 (-0.007, 0.008) | 0.91 | 0.0001 (-0.008, 0.008) | 0.98 | - | - |
| CBF x CA-4 | - | - | 0.006 (-0.002, 0.01) | 0.13 | 0.005 (-0.002, 0.013) | 0.17 | - | - |
| Covariates | | | | | | |  | |
| Age | 0.058 (-0.07, -0.05) | *** | -0.053 (-0.06, -0.04) | *** | -0.057 (-0.07, -0.05) | *** | -0.057 (-0.07, -0.05) | *** |
| Sex | 0.31 (-0.5, -0.13) | ** | -0.35 (-0.53, -0.17) | *** | -0.315 (-0.5, -0.13) | ** | -0.32 (-0.5, -0.14) | *** |
| eTIV | 0.33 (0.24, 0.42) | <0.001 | 0.33 (0.24, 0.42) | *** | 0.3 (0.24, 0.43) | *** | 0.34 (0.24, 0.43) | <*** |

.*** (p<0.001), ** (p<.01) and * (*p* < .05) indicate significant differences.

**Supplementary Table 7:** Fully adjusted models showing the estimates and 95% confidence intervals of the relationship between arterial stiffness (Cf-PWV) at Wave 3 and hippocampal subfield volumes (Panel A), between CBF and subfield volumes (Panel B), these two relationships mutually adjusted (Panel C) and the interaction between PWV-W3, CBF and the hippocampal subfield volumes (Panel D).

|  | Panel A: Relationship between PWV-W3 and subfield volumes | | Panel B: Relationship between CBF and subfield volumes | | Panel C: These relationships (PWV and subfield volumes; CBF and subfield volumes), mutually adjusted | | Panel D: Interaction between PWV-W3, CBF and subfield volumes | |
| --- | --- | --- | --- | --- | --- | --- | --- | --- |
|  | Estimates (95% CI) | p-value | Estimates (95% CI) | p-value | Estimates (95% CI) | p-value | Estimates (95& CI) | p-value |
| PWV-W3 | 0.05 (0.01, 0.1) | * | - | - | 0.052 | * | -0.11 (-0.31, 0.08) | 0.25 |
| CBF | - | - | 0.00003 (-0.01, 0.01) | 0.996 | 0.0008 (-0.01, 0.01) | 0.89 | 0.048 (-0.1, 0.007) | 0.1 |
| PWV-W3 x subiculum | -0.04 (-0.07, -0.01) | ** | - | - | -0.038, | * | - | - |
| P  WV-W3 x DG | -0.036 (-0.07, -0.01) | * | - | - | -0.033 | * | - | - |
| PWV-W3 x CA-3 | 0.01 (-0.04, 0.02) | 0.45 | - | - | 0.01 | 0.45 | - | - |
| PWV-W3 x CA-4 | -0.023 (-0.05, 0.01) | 0.12 | - | - | -0.02 | 0.16 | - | - |
| CBF x subiculum | - | - | 0.009 (0.0016, 0.017) | * | 0.008 (0.002, 0.02) | * | - | - |
| CBF x DG | - | - | 0.008 )0.0005, 0.016) | 0.04 | 0.0072 (0.0005, 0.016) | * | - | - |
| CBF x CA-3 | - | - | 0.0004 (-0.007, 0.008) | 0.91 | 0.0001 (-0.007, 0.008) | 0.98 | - | - |
| CBF x CA-4 | - | - | 0.006 (-0.002, 0.014) | 0.13 | 0.0054 (-0.002, 0.01) | 0.17 | - | - |
| PWV-W3 x CBF x subiculum | - | - | - | - | - | - | 0.0007 (-0.003, 0.004) | 0.73 |
| PWV-W3 x CBF x DG | - | - | - | - | - | - | -0.0007 (-0.004, 0.003) | 0.71 |
| PWV-W3 x CBF x CA-3 | - | - | - | - | - | - | -0.006 (-0.01, -0.002) | ** |
| PWV-W3 x CBF x CA-4 | - | - | - | - | - | - | -0.002 (-0.005, 0.002) | 0.44 |
| Covariates | | | | | | |  | |
| Age | -0.067 (-0.079, -0.056) | *** | -0.06 (-0.07, -0.05) | * | -0.067 (-0.07, -0.05) | *** | -0.07 (-0.08, -0.05) | *** |
| Sex | -0.24 (-0.43, -0.05) | * | -0.27 (-0.46, -0.08) | ** | -0.24 (-0.46, -0.08) | * | -0.25 (-0.44, -0.06) | * |
| eTIV | 0.34 (0.25, 0.44) | *** | 0.35 (0.25, 0.44) | *** | 0.35 (0.25, 0.44) | *** | 0.35 (0.26, 0.45) | *** |
| Education (high level) | -0.17 (-0.37, 0.03) | 0.1 | -0.16 (-0.36, 0.04) | 0.13 | -0.17 (-0.36, 0.04) | 0.11 | -0.16 (-0.36, 0.037) | 0.12 |
| CVD conditions | -0.002 (-0.16, 0.15) | 0.99 | -0.013 (-0.17, 0.14) | 0.87 | -0.004 (-0.17, 0.14) | 0.96 | 0.002 (-0.15, 0.16) | 0.98 |
| Systolic BP | 0.009 (0.003, 0.02) | ** | 0.01 (0.004, 0.02) | ** | 0.009 (0.004, 0.016) | ** | 0.009 (0.003, 0.016) | ** |
| Diastolic BP | -0.012 (-0.02, -0.002) | 0.02 | -0.012 (-0.02, -0.002) | * | -0.012 (-0.02, -0.002) | 0.03 | -0.012 (-0.02, -0.002) | * |
| Antihypertensives | 0.14 (-0.07, 0.35) | 0.21 | 0.138 (-0.08, 0.35) | 0.22 | 0.15 (-0.08, 0.35) | 0.18 | 0.13 (-0.09, 0.35) | 0.25 |
| Smoking (present) | -0.06 (-0.39, 0.27) | 0.71 | -0.055 (-0.39, 0.28) | 0.75 | -0.073 (-0.39, 0.28) | 0.67 | -0.06 (-0.39, 0.27) | 0.73 |
| Exercise (high level) | -0.16 (-0.35, 0.03) | 0.11 | -0.15 (-0.34, 0.04) | 0.12 | -0.16 (-0.34, 0.04) | 0.1 | 0.17 (-0.36, 0.02) | 0.09 |
| Alcohol use | 0.027 (-0.23, 0.29) | 0.84 | 0.021 (-0.24, 0.28) | 0.88 | 0.029 (-0.24, 0.28) | 0.83 | 0.03 (-0.23, 0.29) | 0.82 |
| BMI | -0.006 (-0.02, 0.01) | 0.53 | -0.004 (-0.02, 0.01) | 0.64 | -0.004 (-0.02, 0.13) | 0.65 | 0.004 (-0.02, 0.01) | 0.65 |

.*** (p<0.001), ** (p<.01) and * (*p* < .05) indicate significant differences.

**Supplementary Table 8.** Estimates and 95% confidence intervals of the relationship between arterial stiffness (Cf-PWV) at Wave 1 and hippocampal subfield volumes (Panel A), between cf-PWV at wave 1 and subfield volumes, adjusted for CBF (Panel B) and the relationship between cf-PWV at wave 1 and subfield volumes, adjusted for cf-PWV at wave 3 (Panel C).

|  | Panel A: Relationship between cf-PWV-W1 and volumes of subfields | | Panel B: Relationship between cf-PWV-W1 and volumes of subfields, adjusted for CBF | | Panel C: Relationship between cf-PWW-W1 and volumes of subfields, adjusted for cf-PWV-W3 | |
| --- | --- | --- | --- | --- | --- | --- |
|  | Estimates (95% CI) | p-value | Estimates (95% CI) | p-value | Estimates (95% CI) | p-value |
| PWV-W1 | 0.007 (-0.04, 0.05) | 0.76 | 0.006 (-0.04, 0.05) | 0.81 | -0.024 (-0.07, 0.02) | 0.35 |
| CBF | - | - | -0.002 (-0.01, 0.01) | 0.76 | - | - |
| PWV-W3 | - | - | - | - | 0.065 (0.02, 0.11) | 0.009** |
| PWV-W1 x subiculum | -0.038 (-0.07, -0.01) | 0.02* | -0.04 (-0.07, -0.003) | 0.03* | -0.02 (-0.06, 0.02) | 0.3 |
| PWV-W1 x DG | -0.048 (-0.08, -0.02) | 0.002** | -0.05 (-0.08, -0.01) | 0.004** | -0.039 (-0.8, -0.002) | 0.04* |
| PWV-W1 x CA-3 | -0.007 (-0.04, 0.02) | 0.65 | -0.007 (-0.04, 0.02) | 0.66 | -0.0007 (-0.04, 0.04) | 0.97 |
| PWV-W1 x CA-4 | -0.04 (-0.07, -0.009) | 0.01* | -0.038 (-0.07, -0.007) | 0.02* | -0.038 (-0.07, -0.001) | 0.05* |
| CBF x subiculum | - | - | 0.009 (0.0008, 0.02) | 0.03* | - | - |
| CBF x DG | - | - | 0.007 (-0.0006, 0.01) | 0.07 | - | - |
| CBF x CA-3 | - | - | 0.0003 (-0.007, 0.008) | 0.95 | - | - |
| CBF x CA-4 | - | - | 0.005 (-0.003, 0.01) | 0.2 | - | - |
| PWV-W3 x subiculum | - | - | - | - | -0.032 (-0.07, 0.004) | 0.08 |
| PWV-W3 x DG | - | - | - | - | -0.016 (-0.05, 0.02) | 0.54 |
| PWV-W3 x CA-3 | - | - | - | - | -0.011 (-0.05, 0.02) | 0.54 |
| PW-W3 x CA-4 | - | - | - | - | -0.004 (-0.04, 0.03) | 0.83 |
| Covariates | | | | | | |
| Age | -0.05 (-0.06, -0.04) | <0.001*** | -0.05 (-0.06, -0.04) | <0.001*** | -0.06 (-0.07, -0.04) | <0.001*** |
| Sex | -0.36 (-0.54, -0.18) | <0.001*** | -0.36 (-0.58, -0.18) | <0.001*** | -0.32 (-0.5, -0.14) | <0.001*** |
| eTIV | 0.32 (0.23, 0.42) | <0.001*** | 0.32 (0.23, 0.42) | <0.001*** | 0.33 (0.24, 0.42) | <0.001*** |

**Supplementary Table 9:** Fully adjusted models showing the estimates and 95% confidence intervals of the relationship between arterial stiffness (Cf-PWV) at Wave 1 and hippocampal subfield volumes (Panel A), between cf-PWV at wave 1 and subfield volumes, adjusted for CBF (Panel B) and the relationship between cf-PWV at wave 1 and subfield volumes, adjusted for cf-PWV at wave 3 (Panel C).

|  | Panel A: Relationship between cf-PWV-W1 and volumes of subfields | | Panel B: Relationship between cf-PWV-W1 and volumes of subfields, adjusted for CBF | | Panel C: Relationship between cf-PWW-W1 and volumes of subfields, adjusted for cf-PWV-W3 | |
| --- | --- | --- | --- | --- | --- | --- |
|  | Estimates (95% CI) | p-value | Estimates (95% CI) | p-value | Estimates (95% CI) | p-value |
| PWV-W1 | -0.002 (-0.05, 0.04) | 0.94 | -0.05 (-0.08, 0.02) | 0.24 | -0.004 (-0.05, 0.04) | 0.58 |
| PWV-W3 | - | - | 0.063 (0.015, 0.11) | 0.01* | - | - |
| CBF | - | - | - | - | 0.001 (-0.01, 0.01) | 0.9 |
| PWV-W1 x subiculum | -0.038 (-0.07, -0.007) | 0.02* | -0.02 (-0.06, 0.02) | 0.3 | -0.035 (-0.07, -0.003) | 0.03* |
| PWV-W1 x DG | -0.05 (-0.08, -0.02) | 0.002** | -0.04 (-0.08, -0.002) | 0.04* | -0.05 (-0.08, -0.01) | 0.004** |
| PWV-W1 x CA-3 | -0.007 (-0.04, 0.02) | 0.65 | -0.001 (-0.04, 0.04) | 0.97 | -0.007 (-0.04, 0.02) | 0.66 |
| PWV-W1 x CA-4 | -0.04 (-0.07, -0.01) | 0.01* | -0.038 (-0.07, -0.001) | 0.05* | -0.038 (-0.07, -0.01) | 0.02* |
| PWV-W3 x subiculum | - | - | -0.032 (-0.07, 0.004) | 0.08 | - | - |
| PWV-W3 x DG | - | - | -0.02 (-0.05, 0.02) | 0.38 | - | - |
| PWV-W3 x CA-3 | - | - | -0.01 (-0.05, 0.02) | 0.54 | - | - |
| PWV-W3 x CA-4 | - | - | -0.004 (-0.04, 0.03) | 0.83 | - | - |
| CBF x subiculum | - | - | - | - | 0.01 (-0.001, 0.016) | 0.03* |
| CBF x DG | - | - | - | - | 0.007 (-0.001, 0.015) | 0.07 |
| CBF x CA-3 | - | - | - | - | 0.0002 (-0.01, 0.008) | 0.95 |
| CBF x CA-4 | - | - | - | - | 0.005 (-0.003, 0.013) | 0.2 |
| Covariates | | | | | | |
| Age | -0.06 (-0.07, -0.05) | <0.001*** | -0.06 (-0.08, -0.05) | <0.001*** | -0.06 (-0.07, -0.05) | <0.001*** |
| Sex | -0.28 (0.47, -0.09) | 0.006** | -0.25 (-0.44, -0.05) | 0.01* | -0.28 (-0.47, -0.09) | 0.006** |
| eTIV | 0.34 (0.25, 0.43) | <0.001*** | 0.34 (0.25, 0.44) | <0.001*** | 0.35 (0.25, 0.44) | <0.001*** |
| Education 3 (high) | -0.17 (-0.37, 0.03) | 0.11 | -0.18 -0.38, 0.016) | 0.08 | -0.16 (-0.36, 0.04) | 0.12 |
| CVD conditions | -0.01 (-0.17, 0.14) | 0.89 | 0.004 (-0.15, 0.16) | 0.96 | -0.01 (-0.17, 0.14) | 0.87 |
| Systolic BP | 0.01 (0.004, 0.017) | <0.001*** | 0.01 (0.004, 0.016) | 0.002** | 0.01 (0.005, 0.02) | <0.001*** |
| Diastolic BP | -0.013 (-0.02, -0.002) | 0.02 | -0.012 (-0.02, -0.002) | 0.02* | -0.01 (-0.02, -0.002) | 0.03* |
| Antihypertensives | 0.13 (-0.09, 0.34) | 0.26 | 0.14 (-0.07, 0.35) | 0.2 | 0.14 (-0.08, 0.35) | 0.23 |
| Smoking | -0.03 (-0.36, 0.3) | 0.87 | -0.04 (-0.37, 0.29) | 0.8 | -0.04 (-0.37, 0.29) | 0.83 |
| Exercise | -0.15 (-0.33, 0.04) | 0.14 | -0.15 (-0.34, 0.03) | 0.12 | -0.15 (-0.4, 0.04) | 0.83 |
| Alcohol use | 0.01 (-0.25, 0.27) | 0.95 | 0.015 (-0.24, 0.27) | 0.91 | 0.01 (-0.25, 0.27) | 0.94 |
| BMI | -0.005 (-0.02, 0.01) | 0.57 | -0.005 (-0.02, 0.012) | 0.62 | -0.004 (-0.02, 0.014) | 0.7 |
